# Methoxyacetic acid exposure in rats induces N-butyrylglycinuria consistent with beta-oxidation impairment

**DOI:** 10.1007/s00204-026-04330-1

**Published:** 2026-04-28

**Authors:** Samuele Sala, Janonna Kadyrov, Andres Bernal, Andres M. Castillo, Preechaya Naraprasertkul, Nadia Paesalasakul, Proud Bekanan, Issariya Dhitsuwon, Thanaporn Kulthawatsiri, Reika Masuda, Manthan Sharma, Jutarop Phetcharaburanin, Bruce D. Car, Jose Ivan Serrano Contreras, John C. Lindon, Julien Wist, Jeremy K. Nicholson, Elaine Holmes

**Affiliations:** 1 Centre for Computational and Systems Medicine, Health Futures Institute, Murdoch University, Harry Perkins Building, Perth, WA6150 Australia; 2 Faculty of Medicine Siriraj Hospital, Mahidol University, 2 Wang Lang Rd, Siriraj, Bangkok Noi, Bangkok, 10700 Thailand; 3 The National Phenome Institute, Office of the President, Khon Kaen University, Khon Kaen, 40002 Thailand; 4 Department of Systems Biosciences and Computational Medicine, Faculty of Medicine, Khon Kaen University, Khon Kaen, 40002 Thailand; 5 Formerly Bristol-Myers-Squibb Company, Princeton, NJ USA; 6 Faculty of Medicine, Institute of Global Health Innovation, Imperial College London, Level 1, Faculty Building, South Kensington Campus, London, SW7 2NA UK; 7 Departamento de Química, Universidad del Valle, Cali, 76001 Colombia; 8 Division of Systems Medicine, Department of Metabolism, Digestion and Reproduction, Imperial College, Burlington Danes Building, Du Cane Road, London, W12 0NN UK; 9 Escuela de Ingeniería de Sistemas y Computación, Universidad del Valle, Cali, 76001 Colombia

**Keywords:** Methoxyacetic acid, MAA, Urine, NMR, Metabolomics, Metabonomics

## Abstract

Methoxyacetic acid (MAA) is a testicular toxin that targets spermatocytes and round spermatids by disrupting mitochondrial function, leading to cellular energy depletion. Male Sprague-Dawley rats were given single oral doses of MAA (150 or 650 mg/kg), resulting in no mortality but transient toxicity signs and modest body weight effects, especially at the higher dose. Histopathology revealed dose- and time-dependent testicular damage, with selective germ cell necrosis by 48 h and extensive germ cell loss, spermatic giant cells, and epididymal inflammation observed in high-dose animals by 168 h. Metabolic analysis using high resolution ^1^H NMR spectroscopy and OPLS-DA identified elevated urinary excretion of *N*-butyryl glycine, a marker of mitochondrial dysfunction and impaired β-oxidation. The persistence of *N*-butyryl glycine and altered energy metabolites up to 168 h indicates sustained mitochondrial stress and disruption of ATP-dependent processes essential for spermatogenesis. Moreover, the close structural similarity between MAA and butyrate raises the possibility that MAA interacts directly with enzymes involved in butyryl-CoA turnover during the terminal steps of β-oxidation in rodents.

**Supplementary Information:**

The online version contains supplementary material available at 10.1007/s00204-026-04330-1.

## Introduction

Understanding the mechanisms underlying testicular toxicity is a priority in reproductive toxicology, given the increasing concern over environmental and pharmaceutical agents that may impair male fertility (Gabrielsen and Tanrikut 2016; Krzastek et al. 2020). The testis is highly susceptible to toxic insult, particularly during the complex, highly regulated, and energy-demanding, process of spermatogenesis (Rotimi et al. 2024). Yet despite advances in histopathological assessment and biomarker discovery, the molecular events driving germ cell loss and testicular dysfunction are incompletely understood. Robust experimental models are necessary to elucidate pathological pathways and support the development of predictive toxicological frameworks (Ramirez et al. 2013).

**Fig. 1:**
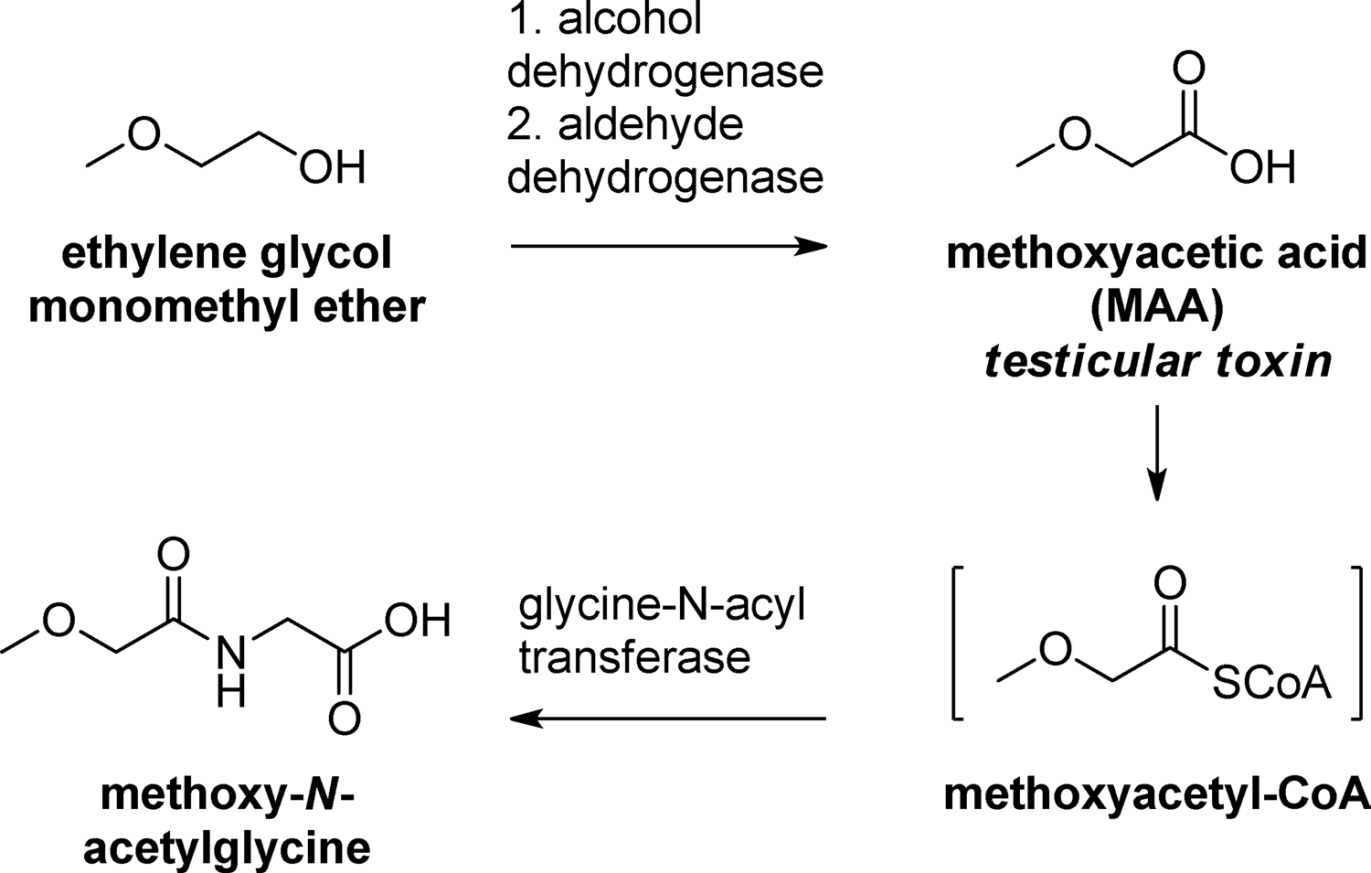
The xenometabolic pathway of ethylene glycol monomethyl ether involves its oxidative biotransformation to the testicular toxin methoxyacetic acid (MAA). MAA is subsequently activated to methoxyacetyl-CoA. From here, the scaffold can undergo conjugation via the action of glycine-N-acyltransferase to form methoxy-*N*-acetyl glycine, which is typically excreted in urine in addition to MAA (Cheever et al. 2001)

Methoxyacetic acid (MAA) (Fig. 1), the major intermediate in the metabolism of the industrial solvent ethylene glycol monomethyl ether (Bagchi and Waxman 2008), is a well-established model compound for investigating testicular toxicity. The German Human Biomonitoring Commission have published reference ranges in human urine with the risk of adverse health effects (HBM II) being set at 1.6 mg MAA/g creatinine (Apel et al. 2017). The half-life for elimination of MAA is estimated to be 12.6 +/- 1.3 h in male rats after a dose of 100 mg/kg (Aasmoe and Aarbakke 1997). MAA has been shown to induce selective degeneration of germ cells, Sertoli cell vacuolation, and disruption of the seminiferous epithelium in a dose-dependent manner, all in the absence of overt systemic toxicity (Miller et al. 1982; Yamazoe et al. 2015). However, reduction in bone marrow cellularity (Miller et al. 1982) has been reported, in addition to teratogenic effects (Brown et al. 1984). Since these toxic effects occur independently of significant changes in circulating gonadotropins or testosterone levels, this would suggest a direct cellular mode of action. Based on in vitro studies in Sertoli-germ cells, the impact of MAA on spermatocytes has been shown to depend on the stage of meiotic maturation with the biggest impact occurring in the dividing and early pachytene stage (Foster et al. 1984). Beattie et al. showed that MAA inhibited stage 3 respiration and the respiratory control ratio in testicular (and hepatic) mitochondria with either succinate or citrate/malate as substrates and inhibition of cytochrome c oxidase (Beattie and Brabec 1986).

Despite its widespread use, key gaps remain in our understanding of MAA-induced testicular toxicity. Emerging evidence suggests that epigenetic dysregulation and intercellular signaling disruptions may contribute to the observed pathological effects. Increased acetylation of core histones have been reported in testis germ cell populations (Wade et al. 2008) and calcium channel blockers such as nifedipine or verapamil can attenuate MAA-induced germ cell apoptosis suggesting that the mechanism of toxicity is at least partially calcium-dependent (Ghanayem and Chapin 1990). Immunocytochemistry studies have shown increased staining of antibodies to various kinases including PKCmu, zeta, and gamma, AKAP220, CaMKII, MLCK, and Src around dying spermatocytes following in vitro exposure to MAA (Jindo et al. 2001). However, the primary molecular targets within Sertoli and germ cells have yet to be fully elucidated, and the temporal sequence of intracellular events leading to germ cell sloughing and death is not well defined.

Metabonomics (also referred to as metabolic phenotyping), (Nicholson et al. 1999), provides a systems-level view of biochemical complexity by capturing the composition, origin, and temporal dynamics of metabolites under various physiological and pathological conditions, reflecting the combined influence of genetic, proteomic, and environmental factors (Nicholson et al. 1999, 2002). Among the analytical platforms employed, ^1^H NMR spectroscopy has become a key platform due to its high reproducibility, quantitative accuracy, and non-destructive nature, supporting applications across biomedicine, nutrition, clinical research, microbiology, and toxicology. The formalization of the field of metabonomics, emerging from a collaboration between Pfizer R&D and Imperial College London, enabled the integration of metabolic profiling with genomics and proteomics to better understand drug efficacy and toxicity (Everett et al. 2013). Since then, NMR- and mass spectrometry-based metabonomic approaches have been widely applied to human and animal models to investigate pharmaceutical, dietary, microbial, and lifestyle influences on metabolism, with multivariate statistical tools playing a central role in data interpretation. A key advancement, pharmacometabonomics (Clayton et al. 2009), extends this framework by predicting individual drug responses from pre- and post-dose metabolic signatures, offering a complementary approach to pharmacogenomics that incorporates both endogenous and environmental variables to improve patient stratification and enable more personalized therapeutic strategies (Everett 2015).

Revisiting historical preclinical data remains a valuable strategy as the field of toxicology transitions toward reduced reliance on animal models. A key example of a large-scale data-set made available for this use is the Consortium on Metabonomic Toxicology (COMET), an industry-academic partnership initiated in 1999 to develop predictive models for organ-specific toxicity based on NMR spectral data from biofluids (Lindon et al. 2005; Kadyrov et al. 2025). Over a three-year period, the COMET project investigated the metabolic effects of 86 acute toxicants and 21 confounding conditions in Sprague-Dawley rats, integrating histopathology and clinical chemistry with metabolic signatures in urine and serum (Ebbels et al. 2007). The resulting datasets informed internal toxicology programs at several pharmaceutical firms, enabling early de-risking of drug candidates and contributing to a number of mechanistic toxicology publications (Beckwith-Hall et al. 1998, 2002; Holmes et al. 2000; Lindon et al. 2003; Yap et al. 2006; Teague et al. 2007; Crunkhorn 2007). Notably, this framework remains relevant to reproductive toxicology, where metabolic disruptions caused by testicular toxicants often raise translational concerns in clinical development. Gaining insight into the biochemical pathways underlying spermatogenesis and predicting such perturbations is essential for both environmental and pharmaceutical toxicologists when assessing the actual risk of specific chemical exposures (Arzuaga et al. 2019).

Here, using both supervised and unsupervised multivariate analyses, we observed a dose-dependent increase in urinary *N*-butyryl glycine in Sprague–Dawley rats following oral gavage administration of MAA. This metabolite was quantified from ^1^H NMR spectra of rodent urine, and its longitudinal excretion profiles are presented. The elimination of short-chain fatty acids (e.g., C4 and C6) is a known marker of mitochondrial overload, as well as incomplete β-oxidation (Koves et al. 2008), and our findings suggest that MAA disrupts this pathway in rats. Given the structural isostericity of methoxyacetyl-CoA with butyryl-CoA, it is plausible that methoxyacetyl-CoA competitively inhibits a terminal-step enzyme in short-chain fatty acid β-oxidation. Such competitive binding could prevent enzymatic processing of butyryl-CoA, causing its accumulation and subsequent conversion to the downstream metabolite *N*-butyryl glycine (Tein et al. 1999).

## Methods

### Ethics

All animal studies were conducted in accordance with the current guidelines for animal welfare (Guide for the Care and Use of Laboratory Animals, 1996) and the procedures used were reviewed and approved by the Institutional Animal Care and Use Committee by the participating company.

## Study design

Male Sprague-Dawley rats aged 6–8 weeks from Charles River Laboratories (Crl: CD(SD)IGS BR) were housed in standard stainless steel wire mesh cages in groups of five and acclimated to the laboratory environment for at least 6 days before being transferred and housed in individual metabolism cages. throughout the studies, and the cages were sterilized daily. All animals were fed powdered Purina chow 5002 (Nestle, Purina, St. Louis, MO) and had free access to water and food throughout the study. The temperature and humidity in the room were maintained at 21 ± 2 °C and 55 ± 10% respectively and continuously monitored. An automatic timing device was used to provide an alternating 12 h light (6 AM-6 PM)/dark cycle. A computer-assisted randomization procedure was used to allocate rats to the metabolism cages. As part of the larger toxicology study, for each test compound, 30 rats were assigned to one of three groups: control, low dose and high dose. The test compound was administered in saline and the route of dosing was selected based on extant literature and the time when animals were given their respective dose was set to zero hours. MAA was administered as a single oral dose (gavage). The following dose levels were selected: **Group 1**: Dose = Control (saline), No. of Rats = 10, Necropsy = 48 h (Group 1 A) or 168 h (Group 1B) after dose; **Group 2**: Dose = 150 mg/kg in saline solution, No. of Rats = 10, Necropsy = 48 h (Group 2 A) or 168 h (Group 2B) after dose; **Group 3**: Dose = 650 mg/kg in saline solution, No. of Rats = 10, Necropsy = 48 h (Group 3 A) or 168 h (Group 3B) after dose.

## Sample collection

Urine samples were collected across an 8-day period including both day and night time points. All animals were placed in metabolism cages 48 h prior to the first urine collection to allow the animals to acclimatise. Urine samples were collected at 16 h pre-dose covering the continuous collection over the 8 h period from − 24 h to − 16, then 0 (− 16–0 h pre-dose), 8 (0–8 h post-dose), 24 (8–24 h post-dose), 48 (24–48 h post-dose), 72 (48–72 h post-dose), 96 (72–96 h post-dose), 120 (96–120 h post-dose), 144 (120–144 h post-dose) and 168 (144–168 h post-dose). Serum samples were collected from all rats via tail vein puncture 24 h after dosing. Half the rats from each group were euthanised at 48 h post-dose and the other half at 168 h post-dose to assess both the acute and chronic effects of each toxin. Serum and tissue samples were collected upon euthanisation, and histopathological examination was conducted. Serum samples were split into two aliquots, one for in-house clinical chemistry analysis and one for proton NMR spectroscopic analysis (performed at Imperial College London). The urine samples were collected into containers with 1 mL of 1% w/v NaN_3_ as a bactericide and maintained at 0 ± 2 °C. On collection, the urine volume was recorded and approximately one-third of each urine sample was transferred to a tube for urinalysis and submitted to clinical pathology. The remainder of the sample was centrifuged for 10 min at ~ 1200 g and subsequently stored at a temperature of − 40 °C until NMR analysis. Urinary volume was recorded, and osmolality was measured using a freezing point depression osmometer. All clinical assays were performed using standard in-house protocols.

## NMR sample preparation

All samples were centrifuged at approx. 1800 g for 5 min to remove any solid debris prior to analysis. Samples were buffered in sodium phosphate and combined with an internal reference (TSP) and anti-bacterial agent (sodium azide). 400 µL of each urine sample was added to 200 µL of sodium phosphate buffer (0.2 M Na_2_HPO_4_, 0.04 M NaH_2_PO_4_ in 80:20 H_2_O: D_2_O, pH 7.4 ± 0.1) containing 1.0 mM TSP (sodium trimethylsilyl propionate-[2,2,3,3-^2^*H*_4_]) and 9 mM NaN_3_.

## NMR data acquisition

^1^H NMR spectra were acquired at 600 MHz and 300 K using a robotic flow-injection system (Bruker Biospin, Karlsruhe, Germany). The standard one-dimensional (1D) experiments with solvent suppression (pp: noesygppr1d) were acquired with 64 scans, 64k data points, relaxation delay of 2.0 s, and a spectral width of 20.0 ppm resulting in a total experiment time of approximately 4 min per sample.

### NMR spectra pre-processing

Spectra were first calibrated to the TSP (trimethylsilyl propionate) singlet (δ_H_ 0.0), followed by the removal of the residual water region (δ_H_ 4.50 to δ_H_ 5.98), and the noise-dominant region (below δ_H_ 0.50 and above δ_H_ 9.50). Additionally the analyses were performed with and without excision of the methoxyacetic acid and methoxy acetyl glycine resonance regions (δ_H_ 3.316 to δ_H_ 3.5 and δ_H_ 3.81 to δ_H_ 4.07). Spectra were baseline corrected and normalised using probabilistic quotient normalisation (PQN). Those pre-processing steps were applied to the rodent urine spectra using the nmr-spectra-processing (“https://github.com/phenological/nmr-spectra-processing”) package in R (version 0.1.4).

### Statistical analysis

Descriptive statistics (median and standard deviation) were summarised for each animal group at baseline and endpoint. Multivariate analyses, including unsupervised principal component analysis (PCA) and supervised orthogonal partial least squares discriminant analysis (OPLS-DA), were conducted on the processed ^1^H NMR spectra using the *mva-plots* package (“https://github.com/phenological/mva-plots”) in order to identify spectral features that differentiate between control and treatment groups. Model performance was assessed using standard metrics of goodness of fit (R²Y) and predictive ability (Q²Y) (Thévenot et al. 2015). Due to the prominence of signals from the test compound methoxyacetic acid and its metabolite methoxyacetyl glycine throughout the 168 h time course, the spectral regions δ_H_ 3.20 to δ_H_ 3.50 and δ_H_ 3.80 to δ_H_ 4.10 were digitally excised and the models recalculated to capture only endogenous metabolic changes. Key discriminatory spectral signals were annotated using a combination of published (Beckwith-Hall et al. 1998; Ebbels et al. 2004; Čermáková et al. 2019), and in-house metabolite databases, and validated by Statistical Total Correlation Spectroscopy (STOCSY), (Cloarec et al. 2005). Correlation matrices were generated using apex intensities of peaks highlighted in loading plots. Univariate analyses were also performed on integrated spectral regions corresponding to identified metabolites to track day-to-day variations across dietary groups.

## Metabolite quantification

Peaks from each metabolite of interest were grouped into Spectral Regions Of Interest (SROI). For each SROI, a list of signals in that region was established with attributes that defined their characteristic patterns (chemical shift, linewidth, multiplicity and scalar coupling). Peaks overlapping features of interest were considered, including unassigned signals. These parameters were used as initial input for a gradient descent optimization (Levenberg-Marquardt) algorithm to minimize the difference between the experimental and the fitted pattern. Fitted peaks were integrated using the full-width at half-maximum approximation.The optimized models were graded using a heuristic based on residuals and correlations between the integrals of different signals of the same analyte. Models with lower grades were visually inspected using a suite of interactive web tools built in JavaScript (Wist 2019). For each metabolite, concentrations were computed based on the best-resolved, highest-intensity signal, and expressed relative to creatinine by calculating the ratio of the corresponding integrals.

## Results

### Clinical chemistry and histopathology

No mortality was observed, though animals exhibited transient signs of toxicity such as ruffled fur and, at the higher dose, mild sedation. Body weight gain was modestly affected, particularly at 650 mg/kg. Clinical biochemistry remained unchanged, and urinalysis showed minimal impact aside from a transient reduction in urinary pH at 48 h p.d, a decrease in ALP at 48 h sustained to 168 h p.d. in the high dose group and an increase in serum phosphorus at 48 h p.d. in both low and high dose groups (Table 1). A notable reduction in testicular weight and testicular size was observed in some high-dose animals at 168 h post-dosing. Microscopic examination revealed dose- and time-dependent testicular and epididymal changes. By 48 h, both dose groups showed selective necrosis of late-stage spermatocytes, while by 168 h, only high-dose animals exhibited significant germ cell loss: particularly of round spermatids and early spermatocytes, alongside increased cellular debris and the presence of spermatic giant cells. Spermatic giant cells were evident in one animal at 650 mg/kg at 48 h and multiple animals at both doses at 168 h. Inflammatory edema in the epididymides was observed in both dose groups at the later time point. The effects on the epididymides were restricted to the 168 h time point when slight to moderate inflammatory edema was observed in some animals from both groups treated with the test article.

**Table 1:** Clinical chemistry and organ weights following methoxy acetate (MAA) exposure

Parameter	Control 48 h *p*.d.	Low dose (150 mg/kg) 48 h *p*.d.	High (650 mg/kg) 48 h *p*.d.	Control 168 h *p*.d.	Low dose (150 mg/kg) 168 h *p*.d.	High (650 mg/kg) 168 h *p*.d.
Testes weight (g)	2.44 ± 0.19	2.68 ± 0.12*	2.35 ± 0.17	2.61 ± 0.17	2.97 ± 0.16*	2.44 ± 0.21*
Body weight (g)	243.84 ± 6.12	251.31 ± 10.22	255.89 ± 19.77	267.20 ± 23.94	295.67 ± 15.68*	282.04 ± 33.24
Urine volume (ml)	11.3 ± 2.8	10.9 ± 2.9	11.0 ± 1.7	12.8 ± 2.4	14.1 ± 2.8	10.9 ± 1.8*
Urine osmolality (mOsm)	1535.2 ± 298.5	1549.3 ± 268.9	1609.9 ± 158.1	1670.8 ± 324.4	1600.8 ± 344.0	1714.6 ± 143.7
Urine pH	7.2 ± 0.2	7.0 ± 0.2**	6.6 ± 0.2**	7.2 ± 0.3	7.0 ± 0.1	7.1 ± 0.3
Urine protein (g/L)	0.8 ± 0.2	0.7 ± 0.2	0.6 ± 0.1**	0.8 ± 0.3	1.0 ± 0.5	0.9 ± 0.2
Urine glucose (nM)	1.2 ± 0.3	1.2 ± 0.3	1.4 ± 0.3	1.3 ± 0.4	1.2 ± 0.3	1.3 ± 0.1
Serum creatinine (uM)	24.40 ± 2.4	20.76 ± 1.13*	20.80 ± 3.30	23.02 ± 2.27	25.02 ± 1.98	23.76 ± 2.35
Serum urea nitrogen (uM)	6650 ± 744.6	5550 ± 475.2	5832 ± 669.8	7254 ± 546.6	6748 ± 640.7	6634 ± 624.2
Serum ALT (IU/L)	51.82 ± 9.91	44.26 ± 5.84	44.82 ± 7.82	58.76 ± 10.07	42.16 ± 2.82*	50.68 ± 2.27
Serum AST (IU/L)	98.92 ± 16.69	90.68I ± 9.57	80.58 ± 7.72	154.74 ± 37.06	118.10 ± 25.55	121.60 ± 17.72
Serum ALP (IU/L)	349.12 ± 79.45	240.44 ± 44.05*	252.06 ± 39.94*	363.76 ± 50.63	336.88 ± 38.54	292.34 ± 29.32*
Serum glucose (mM)	12.29 ± 0.91	10.90 ± 1.29	10.74 ± 0.85*	10.20 ± 4.34	7.96 ± 0.91	8.44 ± 2.07
Serum sodium (mM)	150.50 ± 1.16	148.28 ± 0.59**	148.48 ± 1.53*	150.32 ± 2.60	150.82 ± 1.21	149.70 ± 1.98
Serum potassium (mM)	6.17 ± 0.82	7.67 ± 0.79*	7.32 ± 0.93	7.05 ± 0.61	6.91 ± 0.25	7.65 ± 1.01
Serum calcium (mM)	3.28 ± 0.14	3.27 ± 0.06	3.25 ± 0.10	3.20 ± 0.18	3.13 ± 0.02	3.22 ± 0.15
Serum phosphorous (mM)	3.55 ± 0.14	4.00 ± 0.17**	4.25 ± 0.38**	4.76 ± 0.44	4.30 ± 0.26	4.01 ± 0.21*
Serum albumin (g/L)	43.46 ± 1.62	43.11 ± 0.58	41.73 g ± 1.36	43.42 ± 1.30	43.32 ± 0.85	42.41 ± 0.98
Serum total protein (g/L)	62.08 ± 3.62	60.29 ± 0.78	59.62 ± 1.94	64.01 ± 0.99	62.63 ± 1.34	61.79 ± 3.02
Serum bilirubin (uM)	0.25 ± 0.28	0.22 ± 0.23	0.20 ± 0.24	0.48 ± 0.12	0.34 ± 0.33	0.44 ± 0.25

Significance levels are calculated with respect to the control group at the matched time point; *p < 0.05; ***p < 0.005.

### MAA-induced biochemical alterations in the composition of urine determined by NMR

**Fig. 2:**
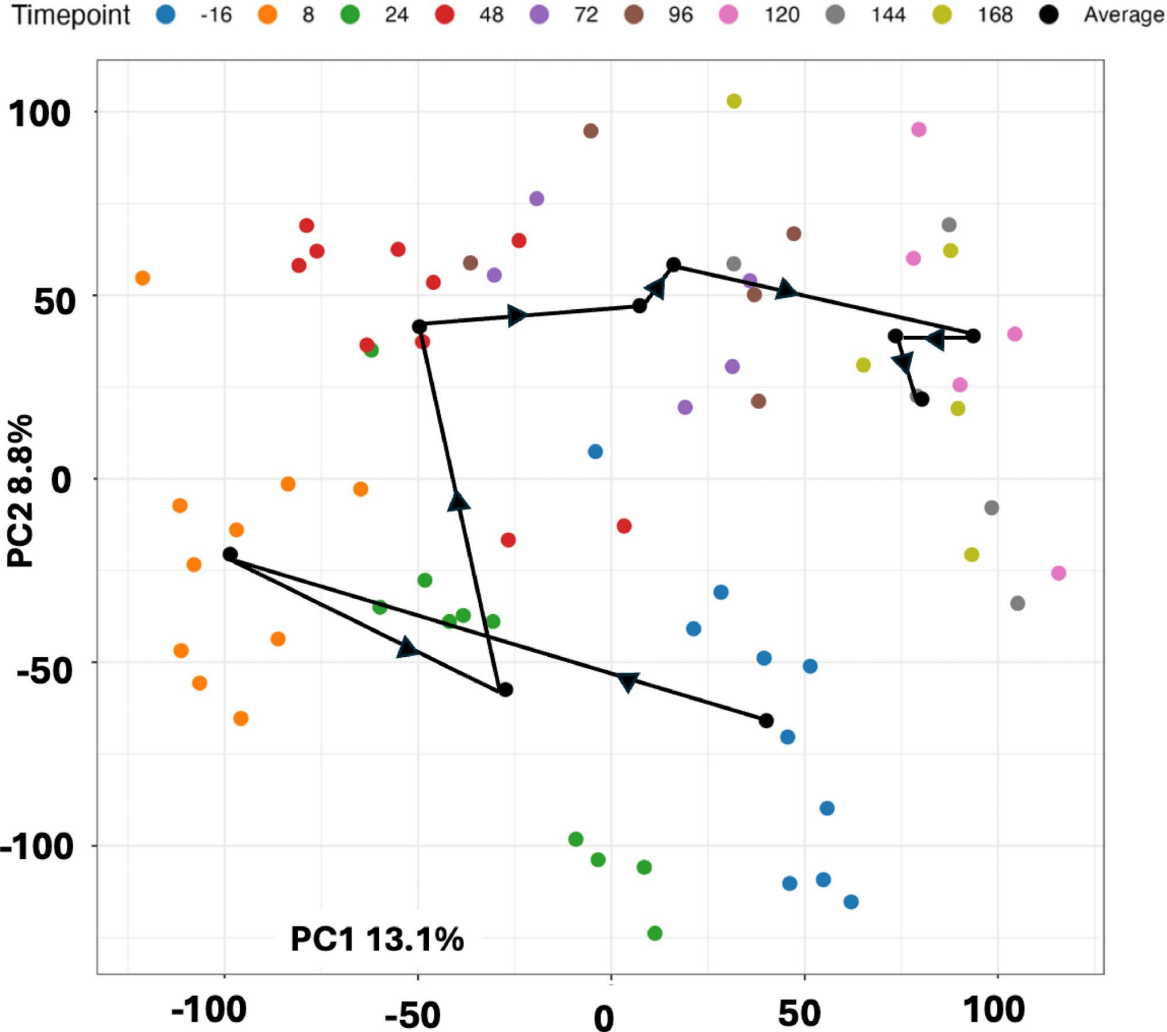
PCA scores plot of all high dose (650 mg/kg) samples following MAA resonance excision coloured by timepoint with average scores for each timepoint represented in black

**Fig. 3:**
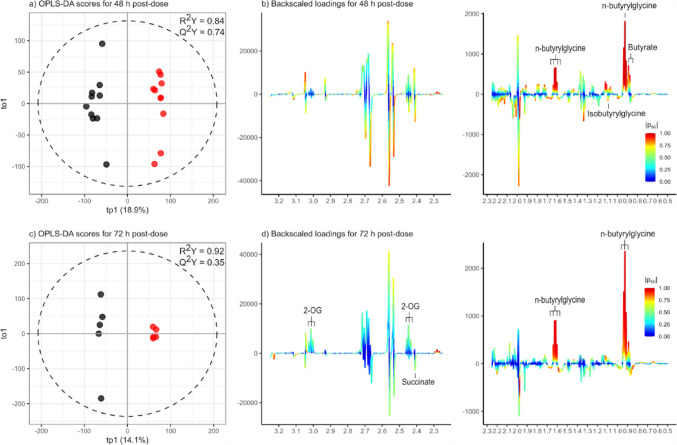
**a** OPLS-DA scores plot of control (black) versus 650 mg/kg (red) at 48 h p.d.;**b** corresponding OPLS-DA loadings plot for 48 h p.d. δ_H_ 0.5–2.3 and 2.25–3.25;**c** OPLS-DA scores plot of control (black) versus 650 mg/kg (red) at 48 h p.d.;**d** corresponding OPLS-DA loadings plot for 72 h p.d. δ_H_ 0.5–2.3 and 2.25–3.25

Analysis of the NMR spectra revealed prominent signals corresponding to methoxyacetic acid (MAA) itself with resonances at δ_H_ 3.37 (s) and δ_H_ 3.87 (s) and its metabolite methoxy-*N*-acetyl glycine with two single peaks at δ_H_ 3.46 and δ_H_ 4.05 as well as a doublet at δ_H_ 3.82 (Figure S1 to S23),(Sumner et al. 1995; Cheever et al. 2001). The signals from MAA persisted for 120 h post dosing (Figure S17). MAA induced a dynamic series of perturbations in the urinary metabolome from as early as 8 h p.d. The PCA trajectory plot, calculated after exclusion of MAA and methoxy-*N*-acetyl glycine, shows an immediate deviation from control samples reaching a maximum at 48 h p.d (Fig. 2). Pairwise OPLS-DA models indicated a series of metabolic perturbations across the timepoints, illustrated here for 48 h and 72 h p.d. (Fig. 2; see supplementary Figures S1 to S23 for other time points). At 8 h p.d. increased 3-hydroxybutyrate, lactate, dimethylamine and dimethylglycine were evident (Supplementary Figure S4). Additionally, resonances from *N*-butyryl glycine, normally below the facile detection level in ^1^H NMR urine spectra were prominent and remained elevated throughout the 168 h time course (see Supplementary Table S1 and Figures S24, S25 and S26 for detailed assignment and excretion profile). Decreased concentrations of 2-oxoglutarate were observed, together with dimethylglycine. By 48 h post-dosing, the urinary metabolic profile showed additional dose-related characteristics including a change in the chemical shift of citrate, consistent with the observed fall in pH at this time point. Hippurate resonances were depleted at 48 h p.d. indicating a likely impact of MAA exposure on the rodent gut microbiome (Figure S8). Additionally, greater excretion of dimethylamine and dimethylglycine was associated with the higher dose of MAA at 48 h p.d. From 72 h onwards, the main characteristic of the dosed animals was the prevalent signals from *N*-butyryl glycine with additional elevation of 2-oxoglutarate. At 168 h p.d., the only perturbations remaining were increased *N*-butyryl glycine, 2-oxoglutarate and decreased succinate, all indicative of impaired mitochondrial function (Fig. 3).

### Quantification and longitudinal analysis

**Fig. 4:**
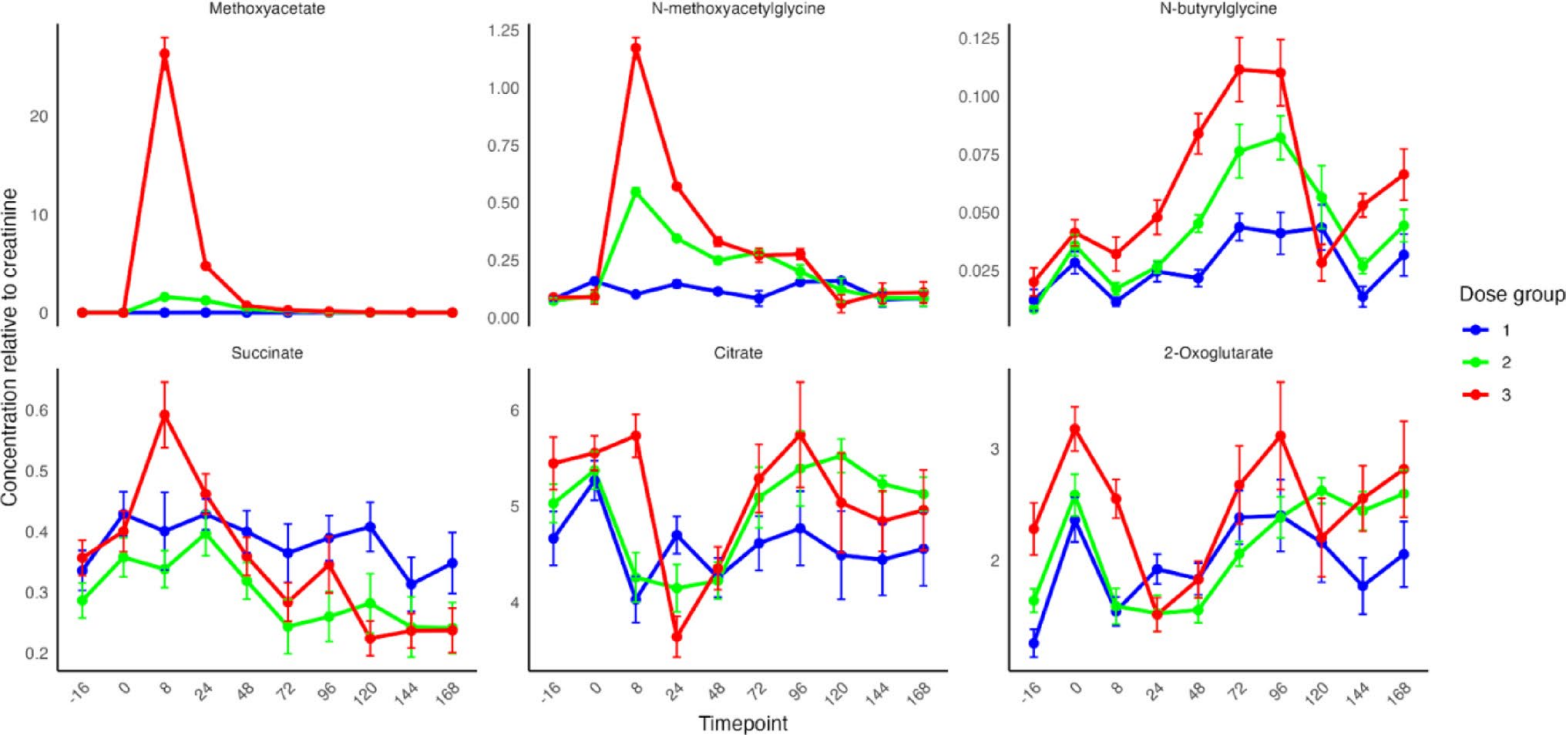
Metabolite profiles across timepoints: Quantification and longitudinal analysis of MAA, methoxy*-N*-acetyl glycine, *N*-butyryl glycine, 2-oxoglutarate, citrate, and succinate following oral dosing with MAA (timepoint = 0), concentration expressed in mM/mM creatinine. Note the effects of diurnal variation visible at timepoints: − 16 h and 8 h

A panel of toxin-responsive metabolites, including *N-*butyryl glycine, 2-oxoglutarate, citrate, and succinate, was quantified using a dedicated line-fitting approach across the time course (Fig. 4, additionally, see supplementary Figs. S27, S28, S29, S30, S31, S32, S33, S34, S35, S36, S37, S38 and S39 for DMA, DMG, hippurate, ketoleucine, alanine, acetate, taurine, glycine, PAGly, allantoin, formate, trigonelline and *N*-methylnicotinamide quantified excretion profiles). Succinate excretion peaked at 8 h p.d. In contrast, Citrate and 2-oxoglutarate reached their lowest levels at 24 h p.d. but both displayed a delayed increase by 96 h representing an inversion of the trend observed for succinate. *N-*butyryl glycine demonstrated a similarly delayed trajectory, with dose-dependent maximal excretion observed at 96 h p.d. Both MAA and methoxy-*N*-acetyl glycine exhibited early maxima at 8 h p.d., however, methoxy-*N*-acetyl glycine displayed a more sustained excretion profile relative to MAA, which seemingly decayed more rapidly.

## Discussion

The MAA model has proven valuable in investigating non-hormonal mechanisms of testicular injury, including mitochondrial dysfunction (Beattie and Brabec 1986) and oxidative stress (Parajuli et al. 2015). Its consistent phenotypic and histological outcomes have led to its adoption as a benchmark compound in regulatory toxicology and mechanistic studies alike. MAA primarily targets spermatocytes and round spermatids, where it disrupts mitochondrial membrane potential, impairs ATP synthesis, and induces energy depletion (Beattie and Brabec 1986; Foster et al. 1987; Davis et al. 1997). These mitochondrial disturbances are accompanied by activation of intrinsic apoptotic pathways in meiotic and post-meiotic germ cells, as well as alterations in chromatin remodeling that impair nuclear maturation during spermatogenesis (Jindo et al. 2001; Bagchi et al. 2009). Beyond its reproductive toxicity, MAA has also demonstrated immunotoxic, hepatotoxic and teratogenic effects (Miller et al. 1982; Rawlings et al. 1985; Poon et al. 2005).

Signals for MAA and its glycine conjugated metabolite were evident until 120 h p.d., with traces remaining at 168 h. The half-life for elimination of MAA has been estimated to be 12.6 +/- 1.3 h in male rats after a dose of 100 mg/kg (Aasmoe and Aarbakke 1997), which is consistent with our observations in which > 90% of the dose was excreted in the first 24 h, either as MAA itself or the glycine conjugate, methoxy-*N*-acetyl glycine (Sumner et al. 1995).

Proteomic analysis has implicated aberrant protein kinase activity in mediating MAA-induced apoptosis in rat germ cells, while elevated expression of the stress-response protein HSP70 has been linked to limb malformations in mice via apoptosis in embryonic limb buds (Ruyani et al. 2005). Notably, NMR-based studies have shown that metabolic modulators such as acetate and serine can mitigate these effects by diverting metabolism away from toxic intermediates, providing protection against both developmental and testicular toxicity (Sumner et al. 1995). In addition, calcium channel blockers such as nifedipine or verapamil can attenuate MAA-induced germ cell apoptosis suggesting that the mechanism of toxicity is at least partially calcium-dependent (Ghanayem and Chapin 1990).

Metabolically, MAA administration has been shown to decrease lactate concentrations in in vitro Sertoli cell cultures at doses of 5 mM (Williams and Foster 1988). In rodent models, it induces damage to spermatocytes undergoing meiotic maturation and division within 24 h of treatment at doses of 100 mg/kg body weight or higher (Foster et al. 1987). The acute oral administration of methoxyacetic acid to rats is also known to cause the selective and stage-dependent destruction of pachytene spermatocytes at all stages other than early to mid-stage VII(Bartlett et al. 1988), which was consistent with the pathology observed in the current study. Metabolic effects include a reduction in urinary creatine and creatinine 24 h p.d., similar to patterns observed with other testicular toxicants such as cadmium chloride (M E Traina P Fazzi E Urbaniand A Mantovani 1997).

The pathology observed in the current study aligned with a sequelae of time and dose-dependent modulations in the urinary metabolome reflecting disruption of the mitochondria including perturbation of tricarboxylic acid cycle intermediates (2-oxoglutarate, citrate, succinate) and *N-*butyryl glycine, indicative of impaired β-oxidation. Pairwise OPLS-DA analyses over the experimental time-course, as well as quantification of key metabolites to track longitudinal excretion trajectories, showed a persistent increase in urinary excretion of 2-oxoglutarate, indicating a shift in energy metabolism consistent with a delayed or compensatory response to acute MAA exposure (Huergo and Dixon 2015). Concurrently, *N*-butyryl glycine excretion peaked at 72 h to 96 h and remained elevated up to the 168 h timepoint, suggesting ongoing mitochondrial impairment in the rodent model.

The identification of *N*-butyryl glycine is particularly significant, as its urinary excretion is commonly linked to mitochondrial overload or defects in β-oxidation, both of which impair ATP production (Koves et al. 2008). In humans, elevated excretion of this metabolite has been sporadically associated with short-chain acyl-coenzyme A dehydrogenase deficiency, an inborn error of metabolism (Bhala et al. 1995). In rodents, its excretion has also been observed under fasting conditions (Čermáková et al. 2019). Since spermatogenesis, particularly meiosis and spermiogenesis, is a highly energy-dependent process, and reliant on mitochondrial β-oxidation (Brauns et al. 2019), the presence of this metabolite suggests a phenotype consistent with disrupted germ cell maturation, in line with the observed histopathological results of this study. Increased urinary concentrations of 3-hydroxybutyrate, as well as an increase in signals attributed to isobutyryl glycine, were also a feature of the early response to MAA.

**Fig. 5:**
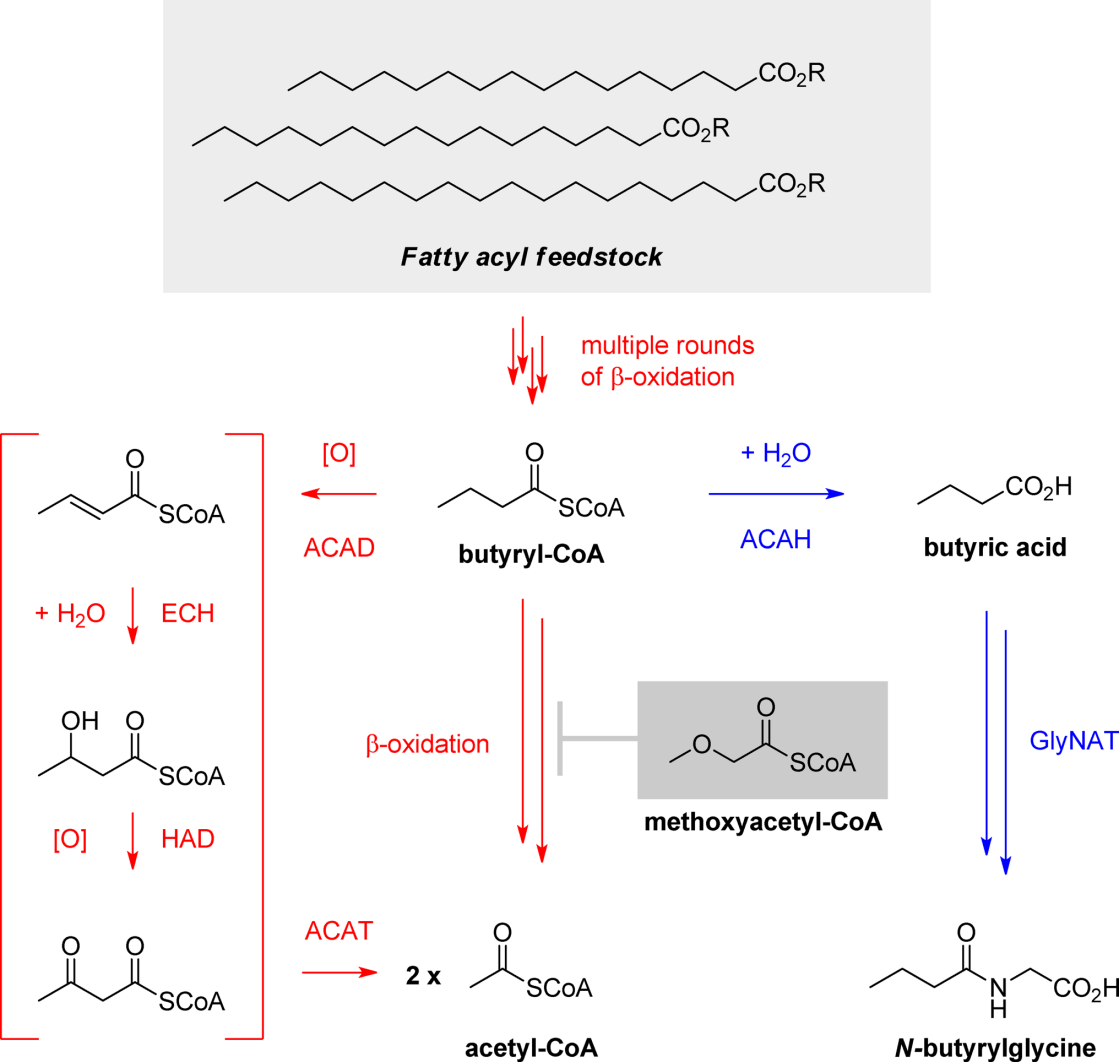
Proposed mechanism for elevated *N*-butyryl glycine excretion following MAA dosing in the sprague dawley rat. As methoxyacetyl-CoA closely resembles butyryl-CoA, it may competitively inhibit enzymes in the final steps of short-chain fatty acid β-oxidation, leading to butyryl-CoA accumulation. The excess butyryl-CoA can then be hydrolyzed to butyric acid, which enters circulation and is ultimately conjugated to glycine in the liver or kidneys, resulting in urinary excretion of N-butyryl glycine. Acronyms: ACAD = acyl-CoA dehydrogenase, ECH = enoyl-CoA hydroxylase, HAD = 3-hydroxyacyl-CoA dehydrogenase, ACAT = 3-ketoacyl-CoA thiolase, ACAH = acyl-CoA hydrolase, GlyNAT = glycine *N*-acyl transferase, + H_2_O = formal hydrolysis, [O] = formal oxidation

Given the structural isostericity of methoxyacetyl-CoA with butyryl-CoA, it is plausible that methoxyacetyl-CoA acts as a competitive inhibitor of an enzyme involved in the terminal steps of short-chain fatty acid β-oxidation, possibly short-chain acyl-CoA dehydrogenase (ACAD) (Swigonová et al. 2009), in line with existing literature (Bhala et al. 1995). Such competitive binding could prevent the enzyme from processing butyyl-CoA, resulting in its accumulation. This excess butyryl-CoA could then be hydrolyzed by acyl-CoA hydrolase to form butyric acid, as has been reported in previous studies (Koves et al. 2008). The freely circulating butyric acid would be expected to cross cell membranes into the bloodstream and subsequently the liver, where it would likely be conjugated to glycine via hepatic glycine N-acyltransferase (Webster et al. 1976; Nandi et al. 1979), ultimately leading to urinary excretion of *N*-butyryl glycine (Fig. 5). We emphasize that this remains a hypothesis and that further biochemical experiments are needed to validate the proposals presented herein. Nevertheless, these findings shed light on the real in-vivo biochemical effects of acute MAA exposure and help define its urinary metabolic phenotype and longitudinal trajectory (Chan et al. 2017). More broadly, this retrospective analysis demonstrates the potential of untargeted metabonomic approaches for novel hypothesis generation.

## Conclusions

Acute MAA exposure in rats elicited a clear temporal sequence of metabolic perturbations, with early increases in urinary excretion of 3-hydroxybutyrate, *N*-butyryl glycine and isobutyrylglycine, and depletion of tricarboxylic acid cycle intermediates, followed by sustained excretion of *N*-butyryl glycine. The persistence of excretion of this metabolite up to 168 h post-dose strongly implicates disruption of β-oxidation, likely through competitive inhibition of short-chain acyl-CoA dehydrogenase by methoxyacetyl-CoA. This mitochondrial impairment aligns with the observed selective degeneration of pachytene spermatocytes and round spermatids, consistent with the dependence of spermatogenesis on intact energy metabolism.

These findings extend current mechanistic understanding of MAA toxicity by integrating metabolic, histopathological, and biochemical evidence, and highlight urinary *N*-butyryl glycine as a potential non-invasive biomarker of mitochondrial dysfunction in the context of testicular injury. The data also support the hypothesis that mitochondrial toxicity underpins both the reproductive and systemic effects of MAA, including its immunotoxic and hepatotoxic actions. Importantly, this metabolic signature offers a translational opportunity for applying NMR-based urinary metabolomics to screen for environmental or occupational toxicants with similar modes of action.

## Supplementary Information

Below is the link to the electronic supplementary material.

Supplementary Material 1
