# Supplementary material for: Methoxyacetic acid exposure in rats induces N-butyrylglycinuria consistent with beta-oxidation impairment
Source: Arch Toxicol. 2026 Apr 28;100(8):3507–18. doi: 10.1007/s00204-026-04330-1 (PMC13379412; doi:10.1007/s00204-026-04330-1)

Supporting Information

Methoxyacetic acid exposure in rats induces N-butyrylglycinuria consistent with beta-oxidation impairment

Samuele Sala (1)*, Janonna Kadyrov (1), Andres Bernal (1), Andres M. Castillo (1, 9), Preechaya Naraprasertkul (2), Nadia Paesalasakul (2), Proud Bekanan (2), Issariya Dhitsuwon (2), Thanaporn Kulthawatsiri (1,3), Reika Masuda (1), Manthan Sharma (1), Jutarop Phetcharaburanin (3,4), Bruce D. Car (5), Jose Ivan Serrano Contreras (6), John C. Lindon (6), Julien Wist (1,6,7), Jeremy K. Nicholson (6), Elaine Holmes (1,8)*

(1) Centre for Computational and Systems Medicine, Health Futures Institute, Murdoch University, Harry Perkins Building, Perth, Australia, WA6150

(2) Faculty of Medicine Siriraj Hospital, Mahidol University, 2 Wang Lang Rd, Siriraj, Bangkok Noi, Bangkok, Thailand, 10700

(3) The National Phenome Institute, Office of the President, Khon Kaen University, Khon Kaen, 40002, Thailand

(4) Department of Systems Biosciences and Computational Medicine, Faculty of Medicine, Khon Kaen University, Khon Kaen, Thailand, 40002

(5) Formerly Bristol-Myers-Squibb Company, Princeton, NJ, USA

(6) Institute of Global Health Innovation, Faculty of Medicine, Imperial College London, Level 1, Faculty Building, South Kensington Campus, London, SW7 2NA, UK.

(7) Departamento de Química, Universidad del Valle, Cali 76001, Colombia.

(8) Division of Systems Medicine, Department of Metabolism, Digestion and Reproduction, Imperial College, Burlington Danes Building, Du Cane Road, London, W12 0NN, UK

(9) Escuela de Ingeniería de Sistemas y Computación, Universidad del Valle, Cali 76001, Colombia.

*Email: SS: Samuele.Sala@murdoch.edu.au

*Email: EH: Elaine.Holmes@murdoch.edu.au

Table of Contents

Historical Histopathological Assessment.. 4

INTRODUCTION.. 4

VIABILITY/MORTALITY.. 4

BODY WEIGHTS.. 5

CLINICAL BIOCHEMISTRY.. 5

urinalysis.. 5

organ weights.. 5

MACROSCOPIC FINDINGS.. 5

MICROSCOPIC FINDINGS.. 5

ASSESSMENT and conclusion.. 6

Table S1: 1H NMR metabolite annotations.. 7

Figure S1: PCA scores plot of all dose groups across all time points prior to MAA resonance excision........ 8

Figure S2: PC1 and PC2 loading plots of all dose groups across all time points showing spectral region δH 0.5-9.5 prior to MAA resonance excision.. 9

Figure S3: OPLS-DA scores plot (blue) versus 650 mg/kg (red) at 8 h post dose prior to MAA resonance excision.......... 10

Figure S4: OPLS-DA loadings plot for 8 h post dose showing spectral regions δH 0.50-2.00, δH 2.00-3.00 and δH 3.00-4.00 prior to MAA resonance excision.. 11

Figure S5: OPLS-DA scores plot (blue) versus 650 mg/kg (red) at 24 h post dose prior to MAA resonance excision........ 12

Figure S6: OPLS-DA loadings plot for 24 h post dose showing spectral regions δH 0.50-9.50.. 13

Figure S7: OPLS-DA scores plot (blue) versus 650 mg/kg (red) at 48 h post dose prior to MAA resonance excision........ 14

Figure S8: OPLS-DA loadings plot for 48 h post dose showing spectral regions δH 0.50-9.50.. 15

Figure S9: OPLS-DA permutation plot for 48 h post dose.. 16

Figure S10: OPLS-DA scores plot (blue) versus 650 mg/kg (red) at 72 h post dose prior to MAA resonance excision...... 17

Figure S11: OPLS-DA permutation plot for 72 h post dose.. 18

Figure S12: OPLS-DA loadings plot for 72 h post dose showing spectral regions δH 0.50-9.50.. 19

Figure S13: OPLS-DA scores plot (blue) versus 650 mg/kg (red) at 96 h post dose prior to MAA resonance excision...... 20

Figure S14: OPLS-DA permutation plot for 96 h post dose.. 21

Figure S15: OPLS-DA loadings plot for 96 h post dose showing spectral regions δH 0.50-9.50.. 22

Figure S16: OPLS-DA scores plot (blue) versus 650 mg/kg (red) at 120 h post dose prior to MAA resonance excision.... 23

Figure S17: OPLS-DA loadings plot for 120 h post dose showing spectral regions δH 0.50-9.50.. 24

Figure S18: OPLS-DA scores plot (blue) versus 650 mg/kg (red) at 144 h post dose prior to MAA resonance excision.... 25

Figure S19: OPLS-DA loadings plot for 144 h post dose showing spectral regions δH 0.50-9.50.. 26

Figure S20: OPLS-DA scores plot (blue) versus 650 mg/kg (red) at 168 h post dose prior to MAA resonance excision.... 27

Figure S21: OPLS-DA loadings plot for 168 h post dose showing spectral regions δH 0.50-9.50.. 28

Figure S22: OPLS-DA scores plot of control (black) versus 650 mg/kg (red) at 24h post dose following MAA resonance excision.. 29

Figure S23: OPLS-DA loadings plot for 24 h post dose showing spectral regions δH 0.50-2.30 and δH 2.25-3.25 following MAA resonance excision.. 30

Figure S24: 1H NMR spectrum of N-butyryl glycine authentic reference standard (600 MHz, 90:10 H2O:D2O)................ 31

Figure S25: JRES spectrum of N-butyryl glycine authentic reference standard (600 MHz, 90:10 H2O:D2O)..................... 32

Figure S26: Stack plot of median spectra for all time points showing spectral region δH 0.80-1.00 and δH 1.50-1.70..... 33

Figure S27: Dimethylamine longitudinal excretion profile.. 34

Figure S28: Dimethylglycine longitudinal excretion profile.. 35

Figure S29: Hippurate Longitudinal excretion profile....36
Figure S30: Ketoleucine Longitudinal excretion profile....37
Figure S31: Alanine Longitudinal excretion profile....38
Figure S32: Acetate Longitudinal excretion profile....39
Figure S33: Taurine Longitudinal excretion profile....40
Figure S34: Glycine Longitudinal excretion profile....41
Figure S35: Phenylacetylglycine Longitudinal excretion profile....42
Figure S36: Allantoin Longitudinal excretion profile....43
Figure S37: Formate Longitudinal excretion profile....44
Figure S38: Trigonelline Longitudinal excretion profile....45
Figure S39: N-methylnicotinamide Longitudinal excretion profile....46

Historical Histopathological Assessment

SUMMARY: ROCHE EXP22 – Methoxyacetic acid

INTRODUCTION

The objective of this study was to characterize the toxicity of methoxyacetic acid and to provide serum, urine and tissue samples for subsequent evaluation of the profile of endogenous metabolites using NMR spectroscopy.

In this study, methoxyacetic acid was administered as a single oral dose (gavage) to male Sprague-Dawley rats. The following dose levels were selected:

Group	Dose	No. of Rats	Necropsy
1	Control (saline)	10	48 h (Group 1A) or 168 h (Group 1B) after dose
2	150 mg/kg	10	48 h (Group 2A) or 168 h (Group 2B) after dose
3	650 mg/kg	10	48 h (Group 3A) or 168 h (Group 3B) after dose

VIABILITY/MORTALITY

There were no deaths.

clinical signs

Ruffled fur was noted on the day after dosing for all animals treated with the test article. This sign persisted for three days in those animals dosed at 650 mg/kg and for two days in those treated at 150 mg/kg.

All animals treated at 650 mg/kg appeared slightly sedated after dosing. This sign was not apparent on subsequent days or in rats treated at 150 mg/kg.

BODY WEIGHTS

Animals treated at 650 mg/kg and, to a lesser extent at 150 mg/kg, showed slight fluctuations in body weight gain after dosing when compared with the body weight development of the control group.

CLINICAL BIOCHEMISTRY

There was no evidence of an effect of treatment with MAA on any of the clinical biochemistry parameters.

urinalysis

With the possible exception of a reduction in pH values observed 8, 24 and 48 hours after dosing in animals treated at 650 mg/kg, urinalysis parameters were considered not to have been affected by treatment with the test article.

organ weights

The reduction in testicular weight recorded for animals killed 168 hours after dosing at 650 mg/kg was attributed to the administration of the test article.

MACROSCOPIC FINDINGS

The only macroscopic finding attributed to the administration of methoxyacetic acid was the reduction in the size of the testes seen in one animal killed 168 hours after dosing at 650 mg/kg.

MICROSCOPIC FINDINGS

Test article related changes were observed in the testes and epididymides.

For the animals killed 48 hours after dosing, the testes showed selective necrosis of single cells, especially the late stage spermatocytes at both 150 and 650 mg/kg. The severity and incidence of the lesion were dose related. At 168 hours after dosing, animals treated at 650 mg/kg, but not 150 mg/kg, showed a loss of germ cells, especially the round spermatids and some spermatocytes in the early stages of the spermatic cycle. An increased amount of cellular debris was also evident in the testes of these animals. Spermatic giant cells were observed in animals treated at 150 mg/kg 168 hours after dosing and in animals treated at 650 mg/kg at both 48 and 168 hours.

The epididymides of several animals treated at 650 and 150 mg/kg showed inflammatory edema 168 hours after dosing.

ASSESSMENT and conclusion

The acute oral administration of methoxyacetic acid to rats is known to cause the selective and stage-dependent destruction of pachytene spermatocytes at all stages other than early to mid-stage VII.

In the present study, pathological changes were induced in the testes and epididymides of rats treated with methoxyacetic acid at 150 and 650 mg/kg.

The effects on the testes were dose-related and showed a progression from single germ cell necrosis, affecting mainly the spermatocytes in stages XIII and XIV, at 48 hours after dosing to loss of germ cells affecting mostly the round spermatids but also the spermatocytes of early stages I to V at 168 hours after dosing. An increased amount of cellular debris was apparent in most rats treated at 650 mg/kg and killed after 48 hours, with a further increase apparent after 168 hours. Spermatic giant cells were evident in one animal at 650 mg/kg at 48 hours and at both doses at 168 hours.

The effects on the epididymides were restricted to the 168 hour time point when slight to moderate inflammatory edema was observed in some animals from both groups treated with the test article.

The study successfully completed the objectives of characterizing the toxicity of the test article and generating serum, urine and tissue samples for the determination of the metabolic profile of the test article by NMR spectroscopy.

Table S1: ^{1}H NMR metabolite annotations

Analyte	Chemical Shifts (Multiplicities)
2-Oxoglutarate	2.45 (t) 3.00 (t)
3-Hydroxybutyrate	4.13 (m) 2.34 (d) 1.19 (d)
Acetate	1.92 (s)
Alanine	1.48 (d) 3.78 (q)
Allantoin	5.39 (s)
Butyrate	0.90 (t) 1.56 (tq) 2.15 (t)
Creatine	3.04 (s) 3.94 (s)
Creatinine	3.05 (s) 4.06 (s)
cis-Aconitic acid	3.12 (d) 5.72 (t)
Citrate	2.54 (d) 2.69 (d)
Dimethylamine	2.73 (s)
Dimethylglycine	2.92 (s) 3.71 (s)
Formate	8.46 (s)
Glycine	3.57 (s)
Hippurate	3.97 (d) 7.56 (m) 7.64 (m) 7.83 (m) 8.50 (br)
Isobutyryl glycine	1.10 (d) 2.55 (hep) 3.74 (d)
Ketoleucine	3.67 (d) 2.10 (m) 0.94 (d)
Lactate	1.33 (d) 4.12 (q)
Methoxyacetic acid	3.37 (s) 3.87 (s)
Methoxy-N-acetyl glycine	3.46 (s) 3.82 (d) 4.05 (s)
Methylamine	2.61 (s)
N-Butyryl glycine	3.76 (d) 2.28 (t) 1.62 (m) 0.92 (t)
N-Methylnicotinamide	4.48 (s) 8.18 (m) 8.90 (d) 8.97 (d) 9.28 (s)
Phenylacetylglycine	3.67 (s) 3.78 (d) 7.37 (m) 7.38 (m) 7.43 (m) 8.01 (br)
Succinate	2.41 (s)
Taurine	3.27 (t) 3.43 (t)
trans-Aconitic acid	6.60 (s) 3.46 (s)
Trigonelline	4.44 (s) 8.09 (m) 8.84 (m) 9.12 (s)

Figure S1: PCA scores plot of all dose groups across all time points prior to MAA resonance excision

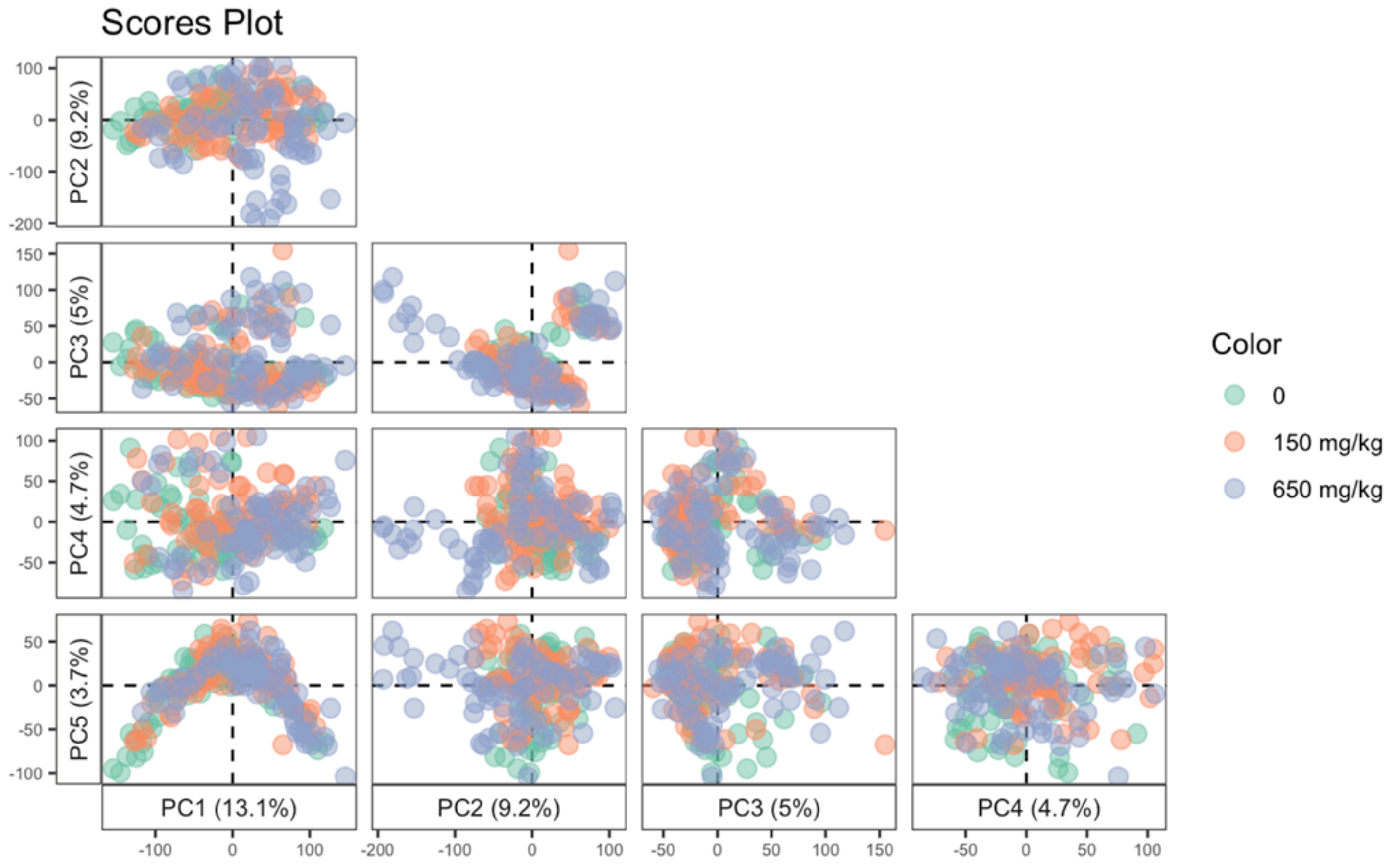

Figure S2: PC1 and PC2 loading plots of all dose groups across all time points showing spectral region δ_H 0.5-9.5 prior to MAA resonance excision

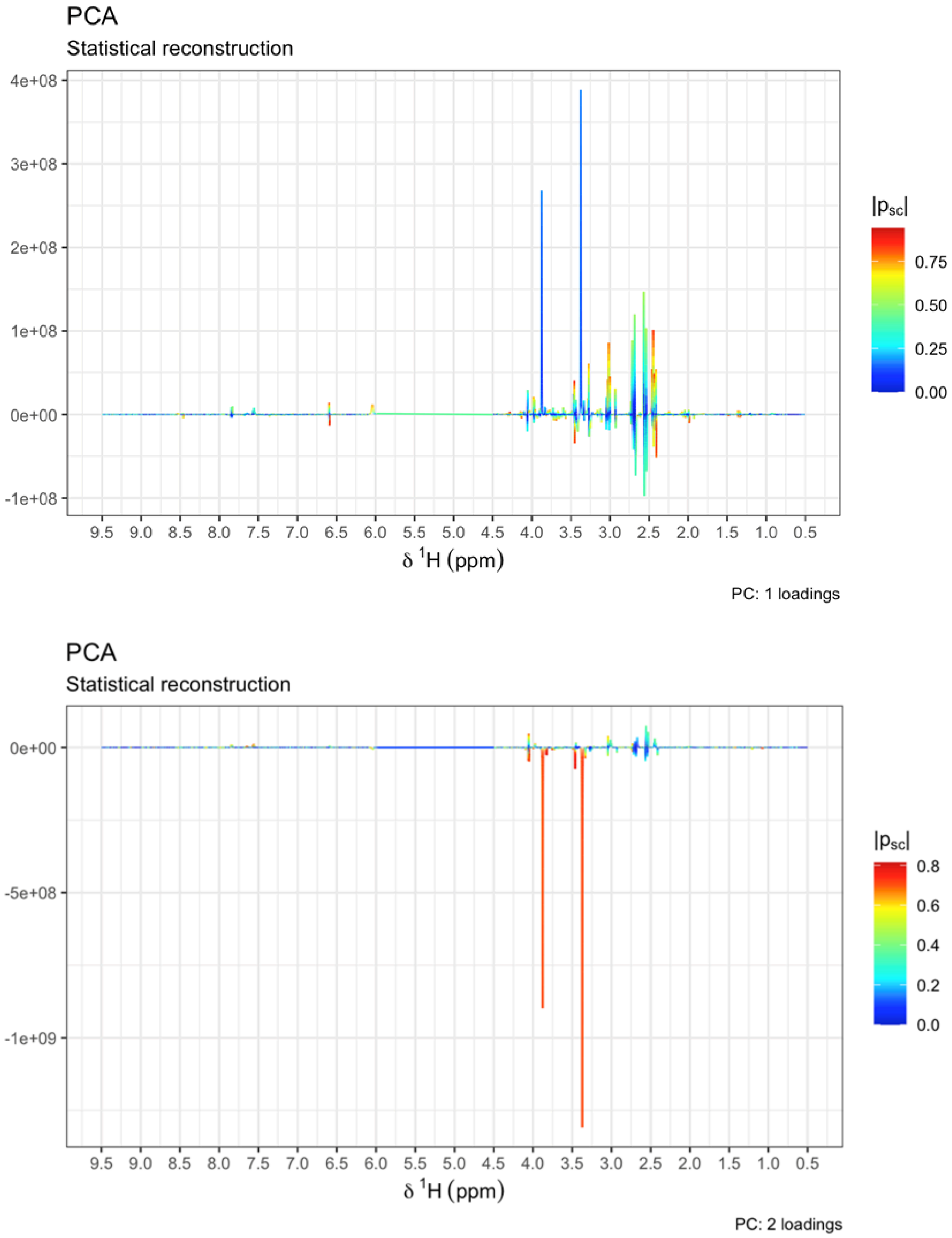

Figure S3: OPLS-DA scores plot (blue) versus 650 mg/kg (red) at 8 h post dose prior to MAA resonance excision

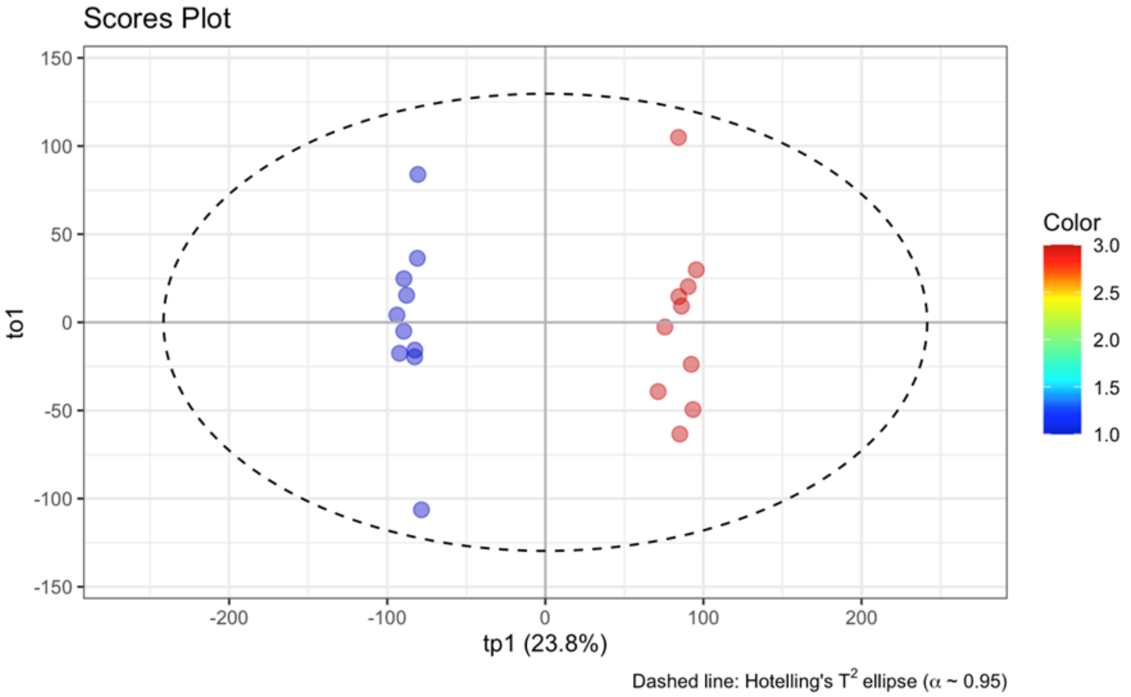

Figure S4: OPLS-DA loadings plot for 8 h post dose showing spectral regions δ_H 0.50-2.00, δ_H 2.00-3.00 and δ_H 3.00-4.00 prior to MAA resonance excision

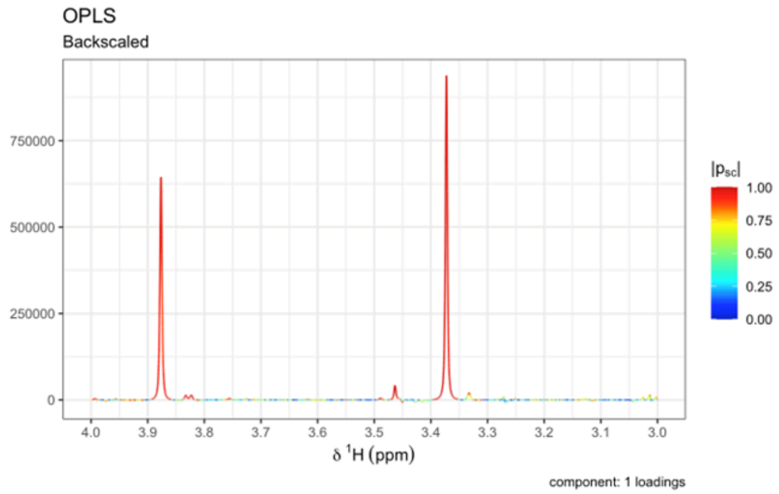

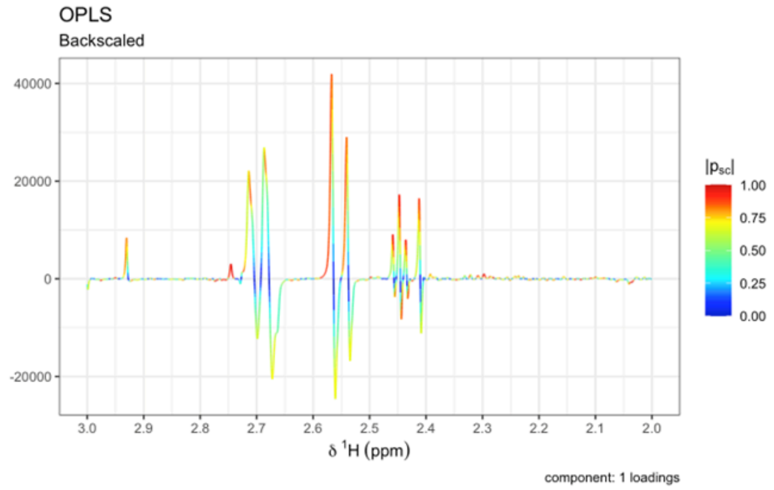

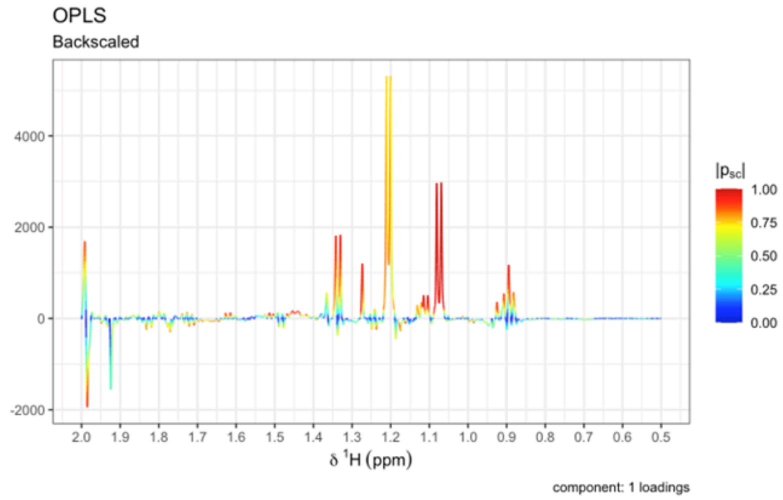

Figure S5: OPLS-DA scores plot (blue) versus 650 mg/kg (red) at 24 h post dose prior to MAA resonance excision

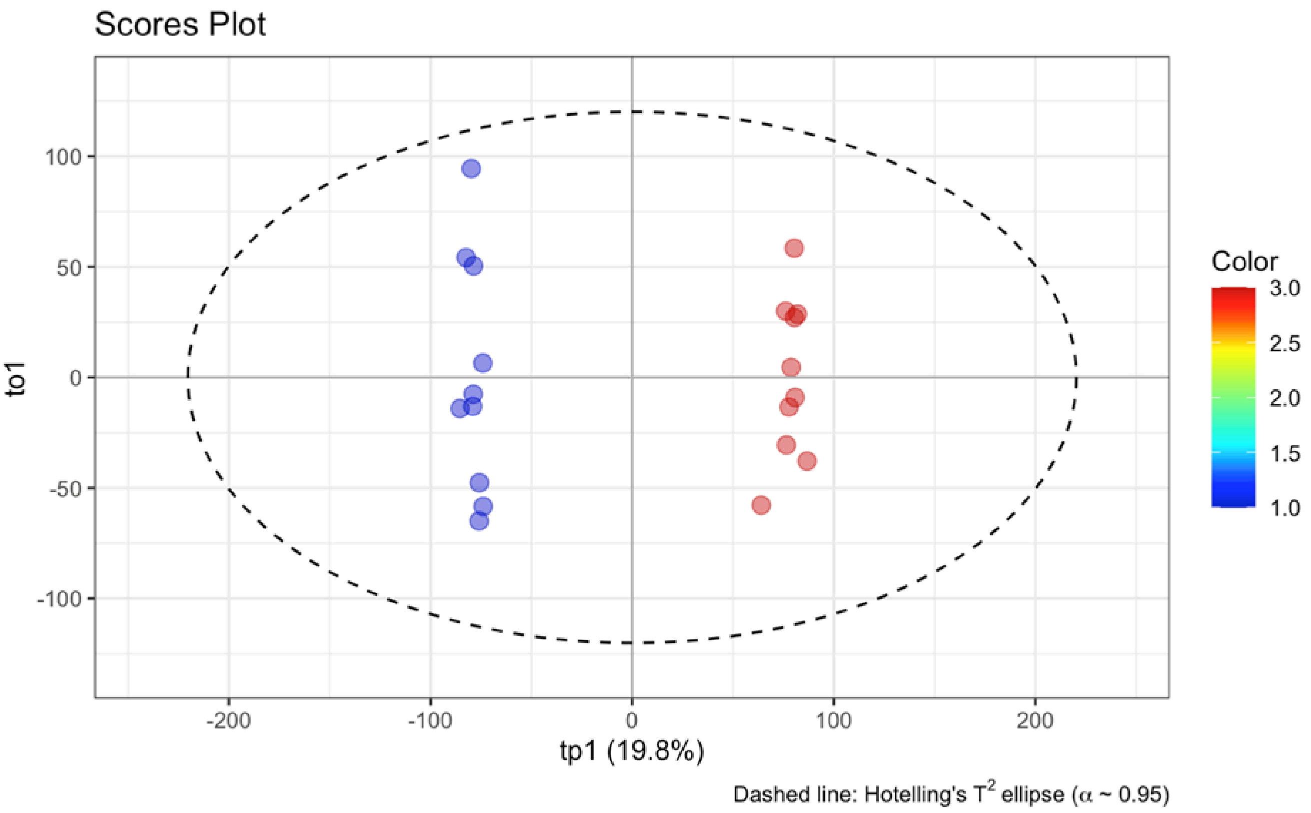

Figure S6: OPLS-DA loadings plot for 24 h post dose showing spectral regions δ_H 0.50-9.50

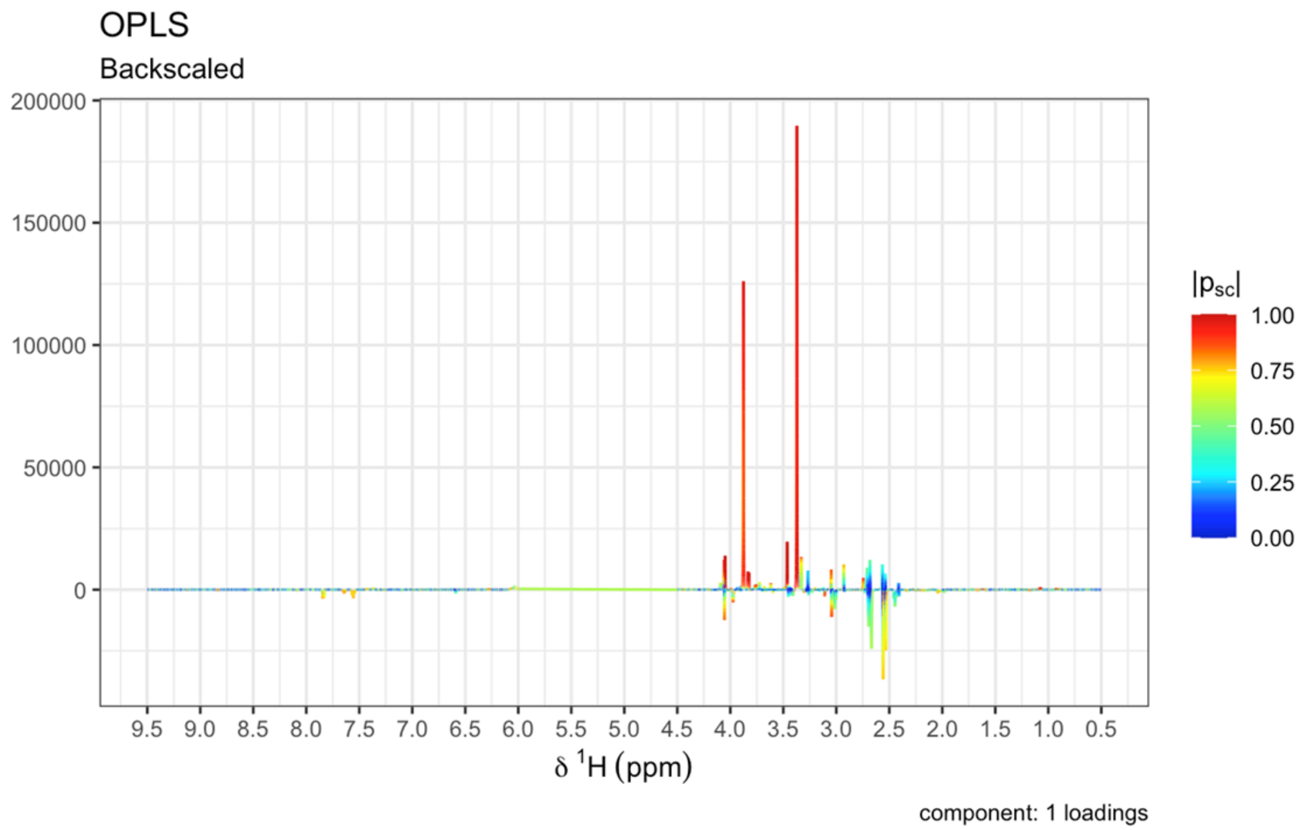

Figure S7: OPLS-DA scores plot (blue) versus 650 mg/kg (red) at 48 h post dose prior to MAA resonance excision

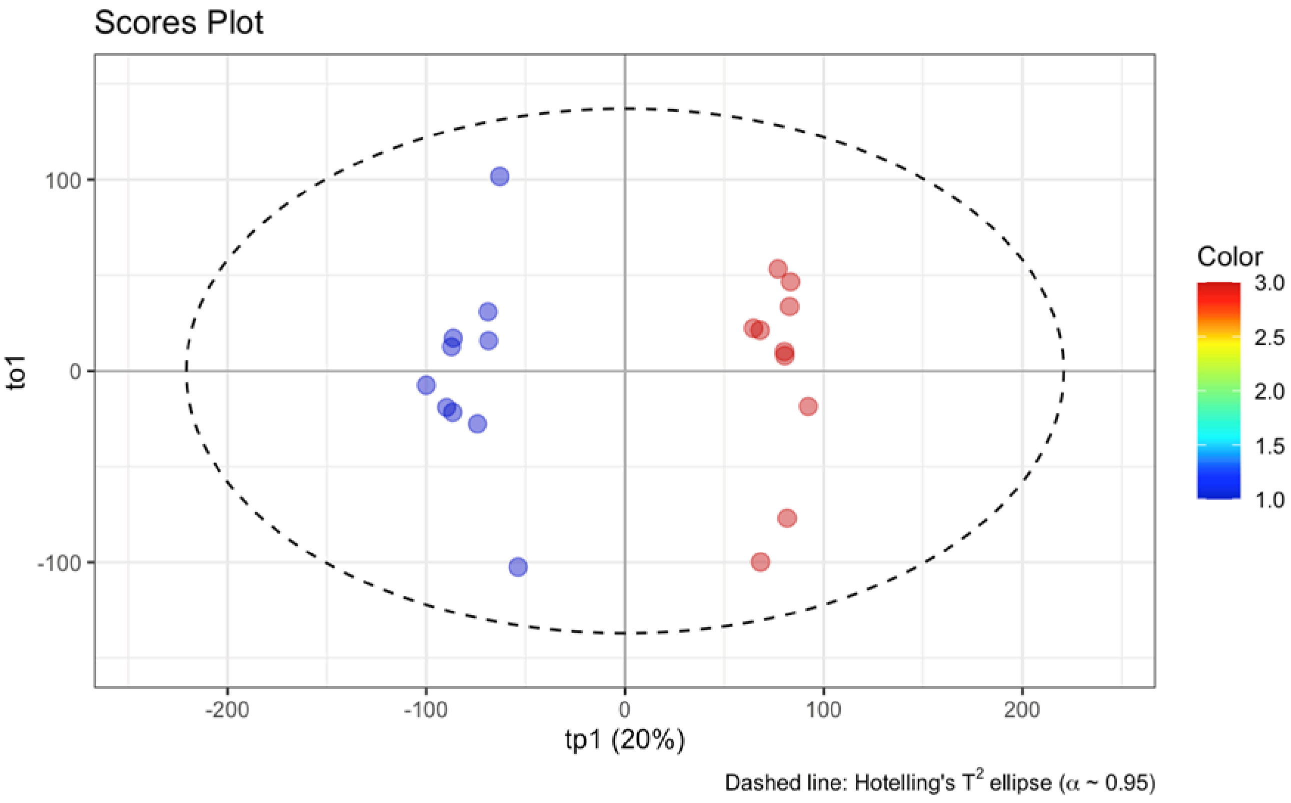

Figure S8: OPLS-DA loadings plot for 48 h post dose showing spectral regions δ_H 0.50-9.50

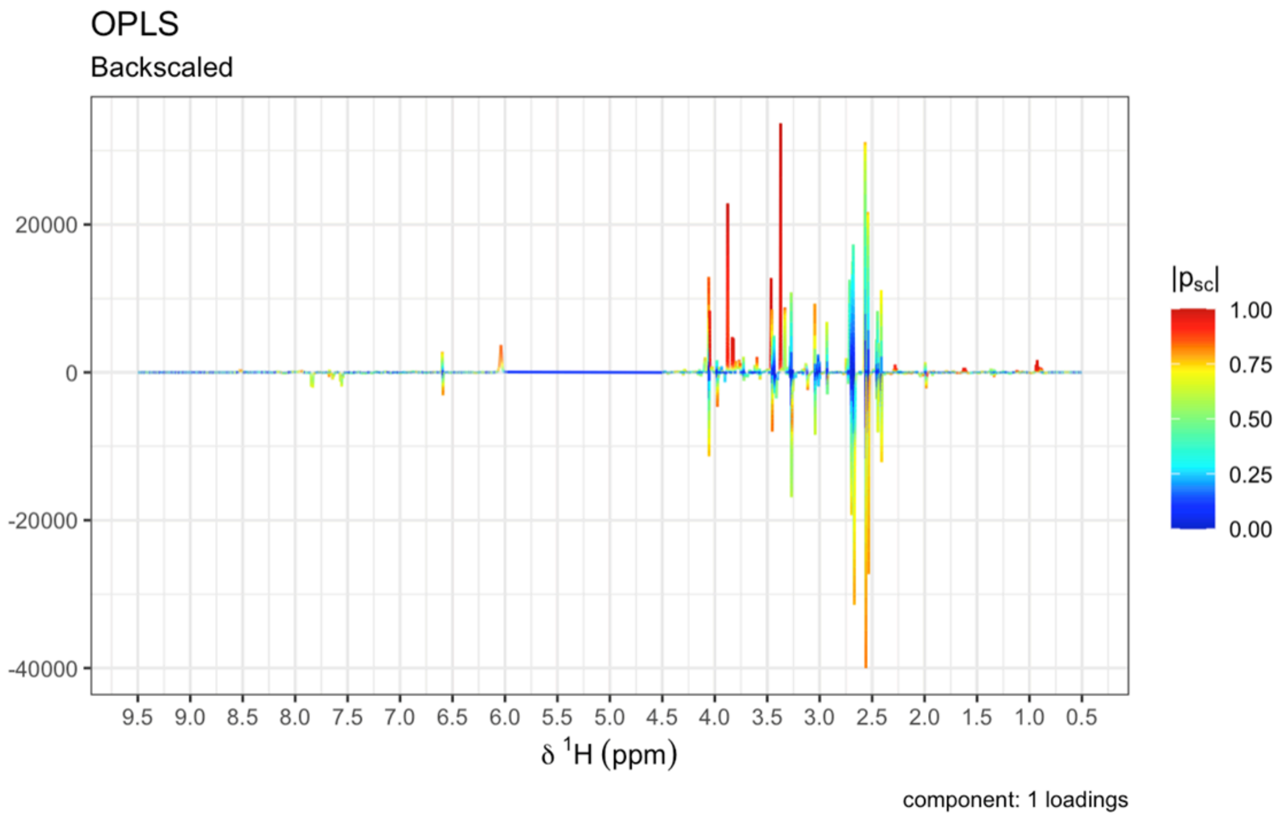

Figure S9: OPLS-DA permutation plot for 48 h post dose

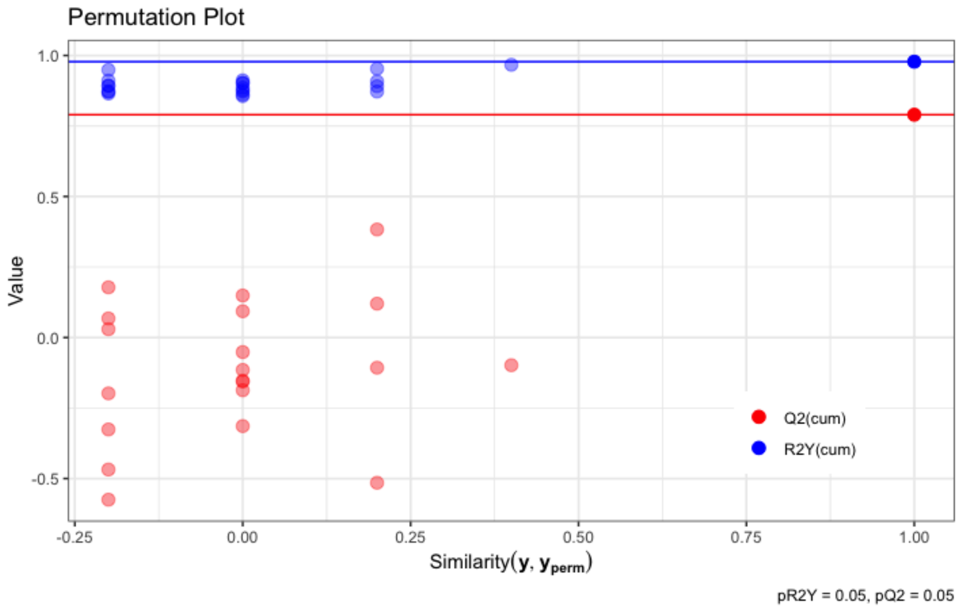

Figure S10: OPLS-DA scores plot (blue) versus 650 mg/kg (red) at 72 h post dose prior to MAA resonance excision

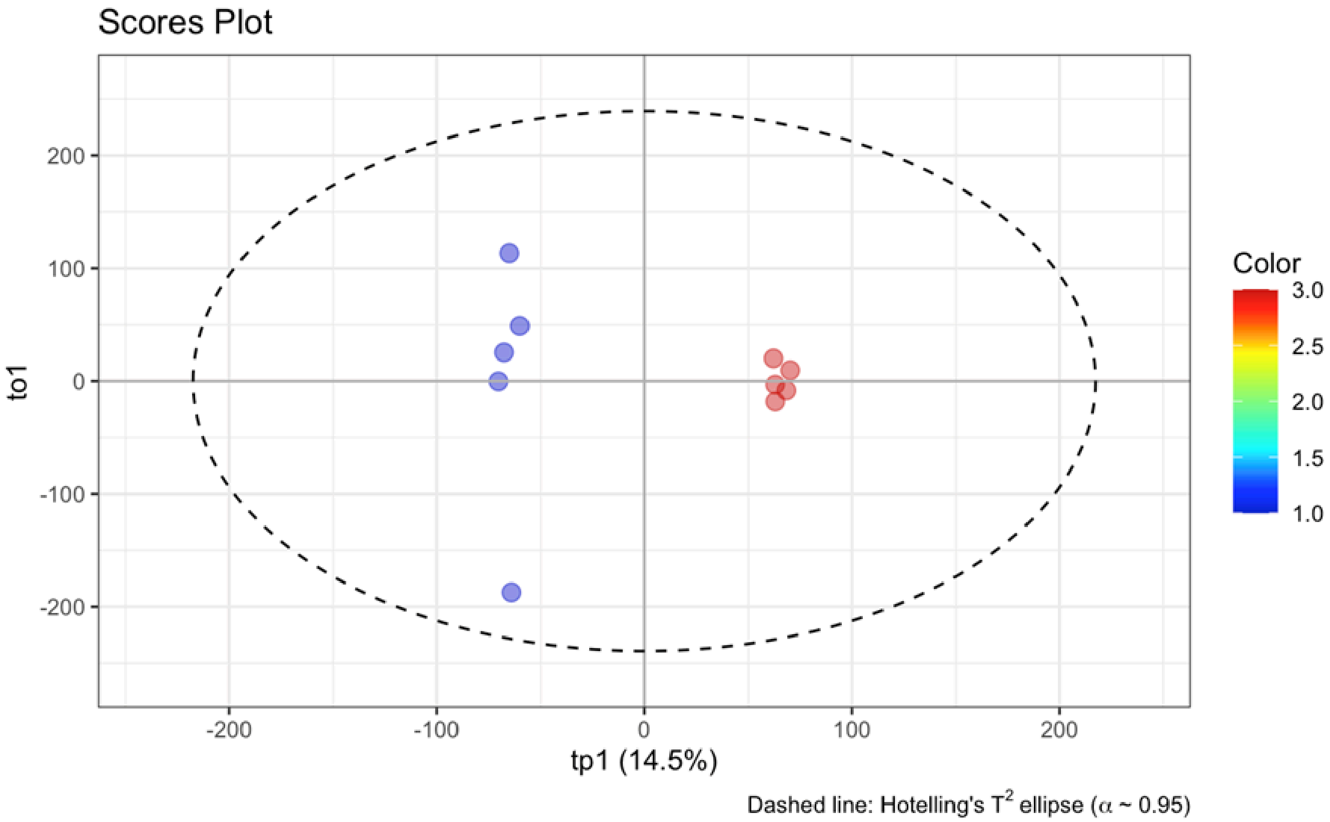

Figure S11: OPLS-DA permutation plot for 72 h post dose

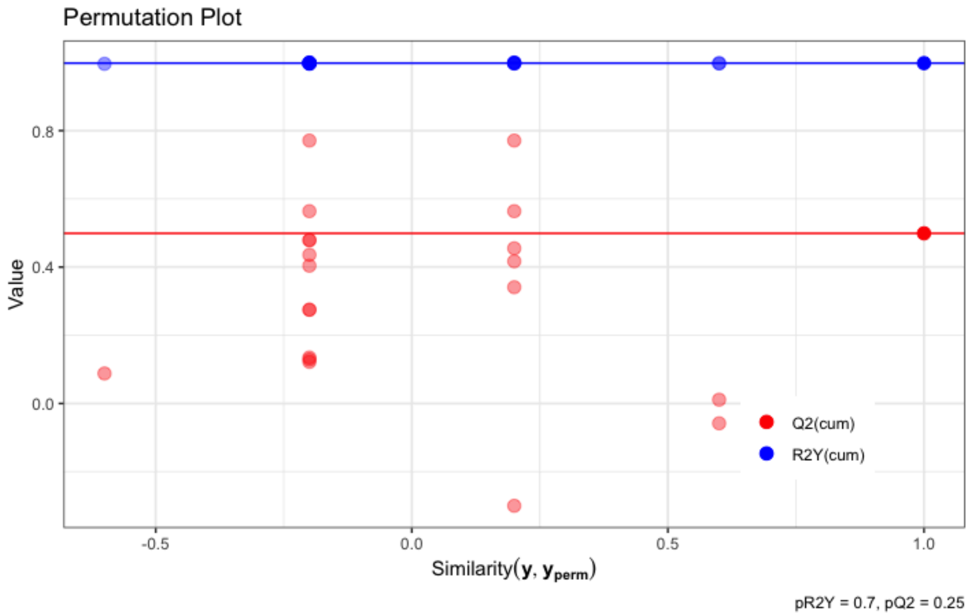

Figure S12: OPLS-DA loadings plot for 72 h post dose showing spectral regions δ_H 0.50-9.50

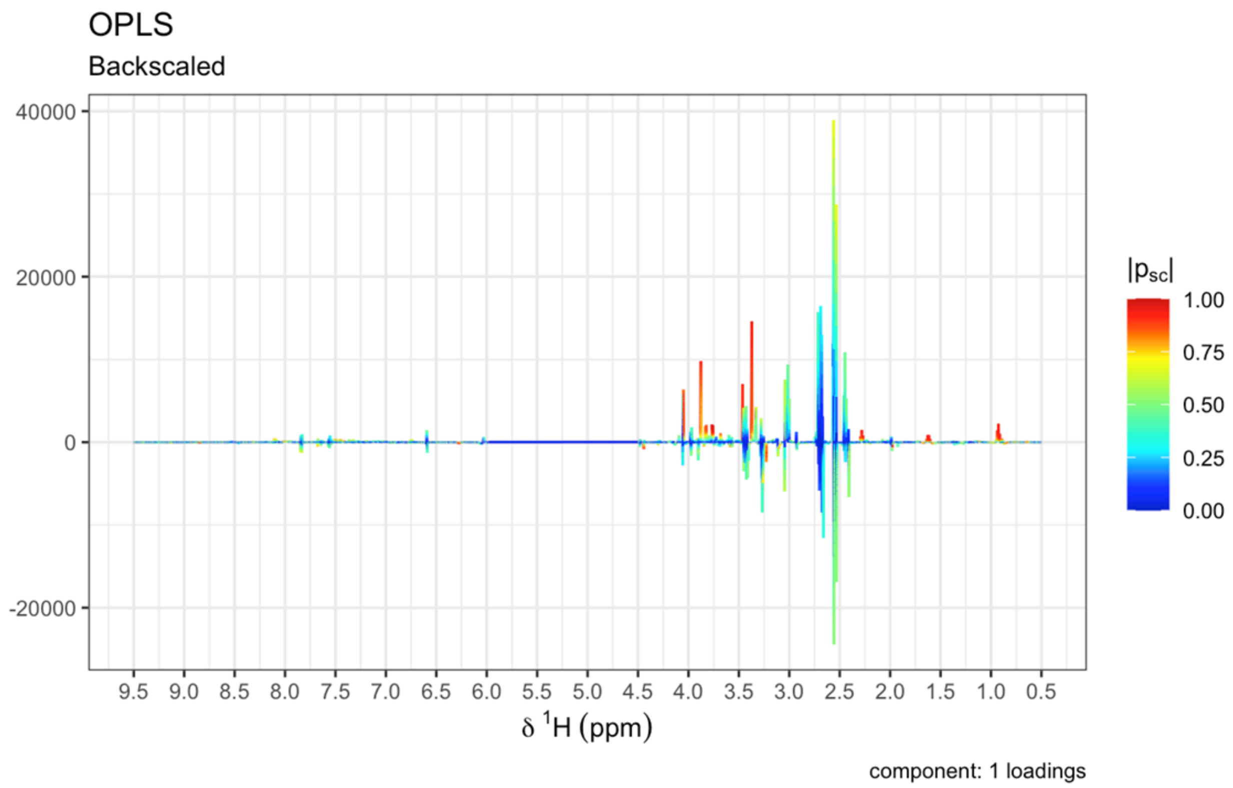

Figure S13: OPLS-DA scores plot (blue) versus 650 mg/kg (red) at 96 h post dose prior to MAA resonance excision

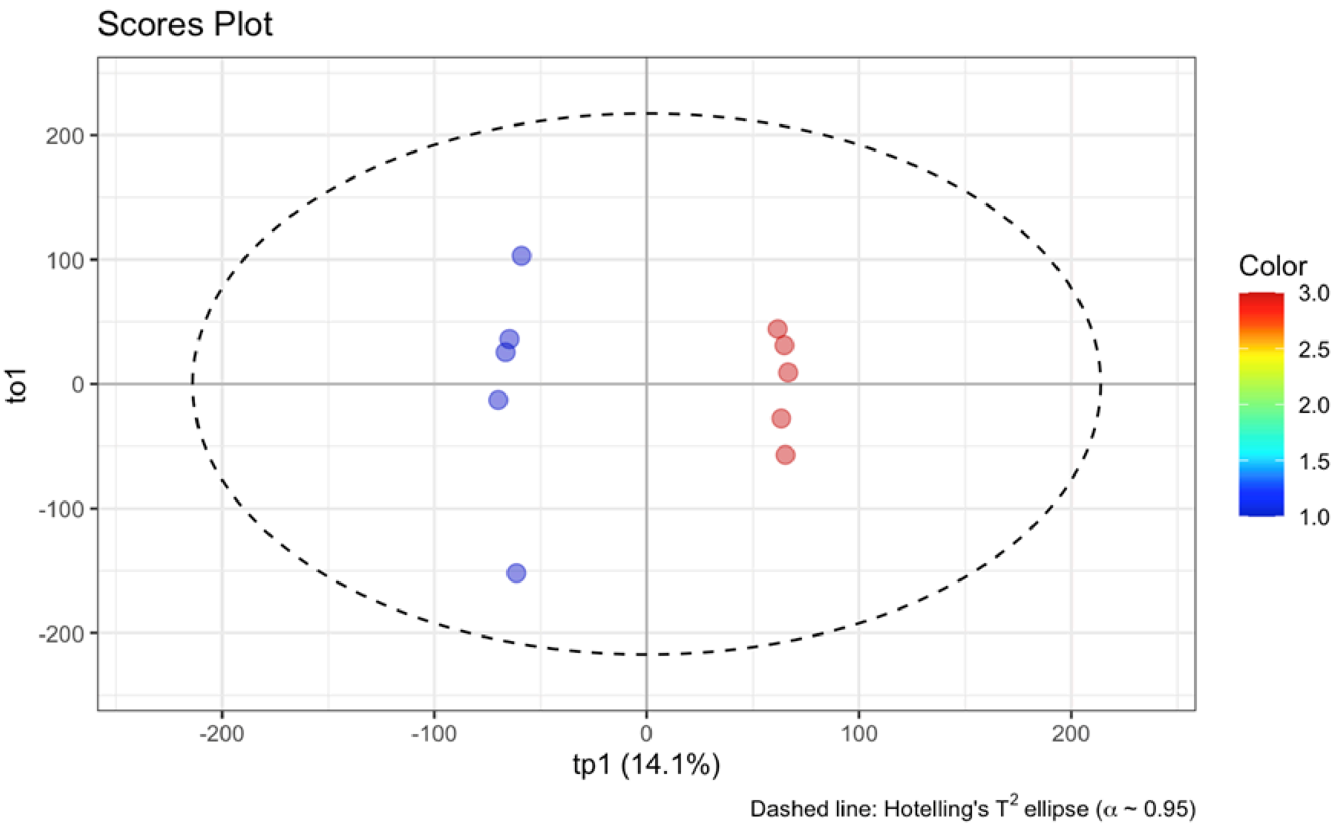

Figure S14: OPLS-DA permutation plot for 96 h post dose

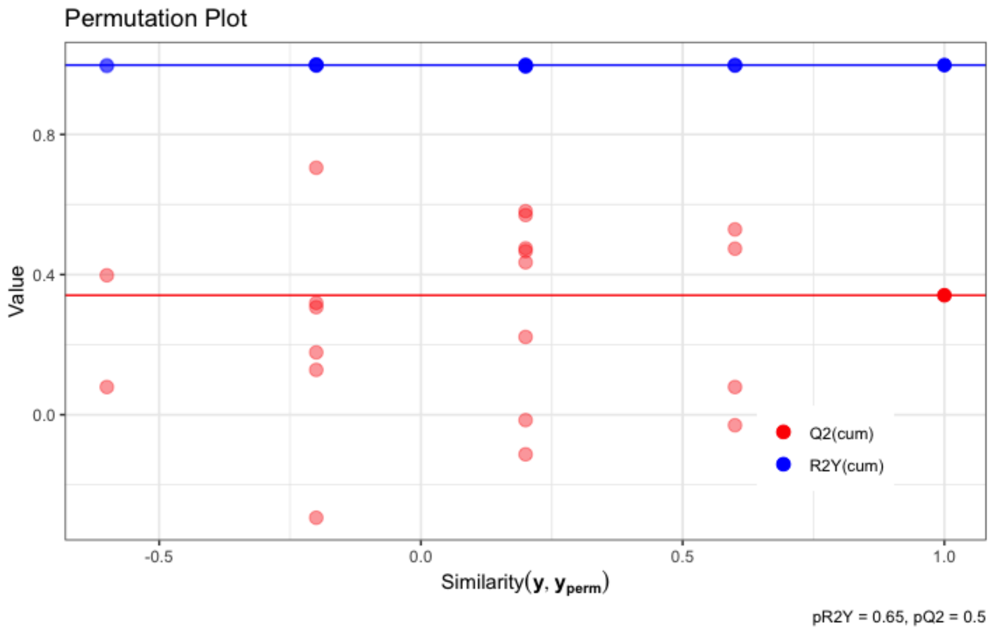

Figure S15: OPLS-DA loadings plot for 96 h post dose showing spectral regions δ_H 0.50-9.50

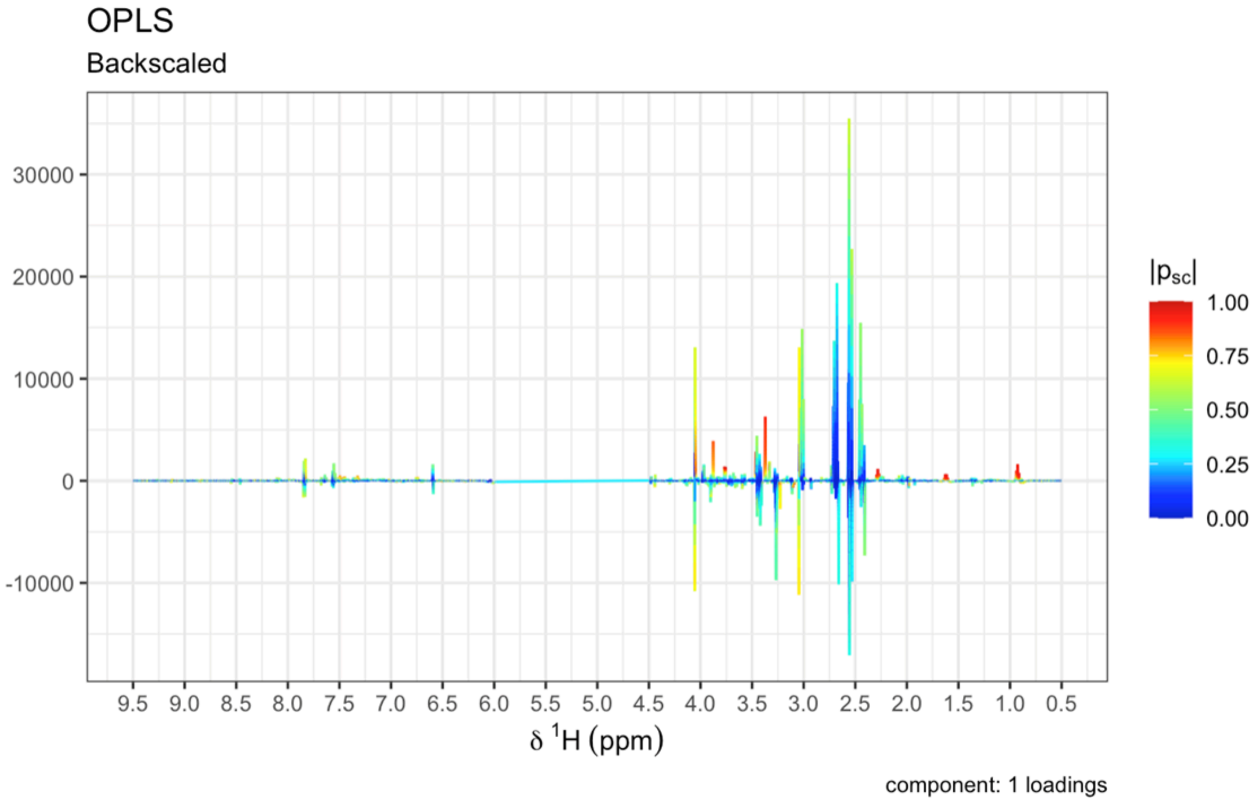

Figure S16: OPLS-DA scores plot (blue) versus 650 mg/kg (red) at 120 h post dose prior to MAA resonance excision

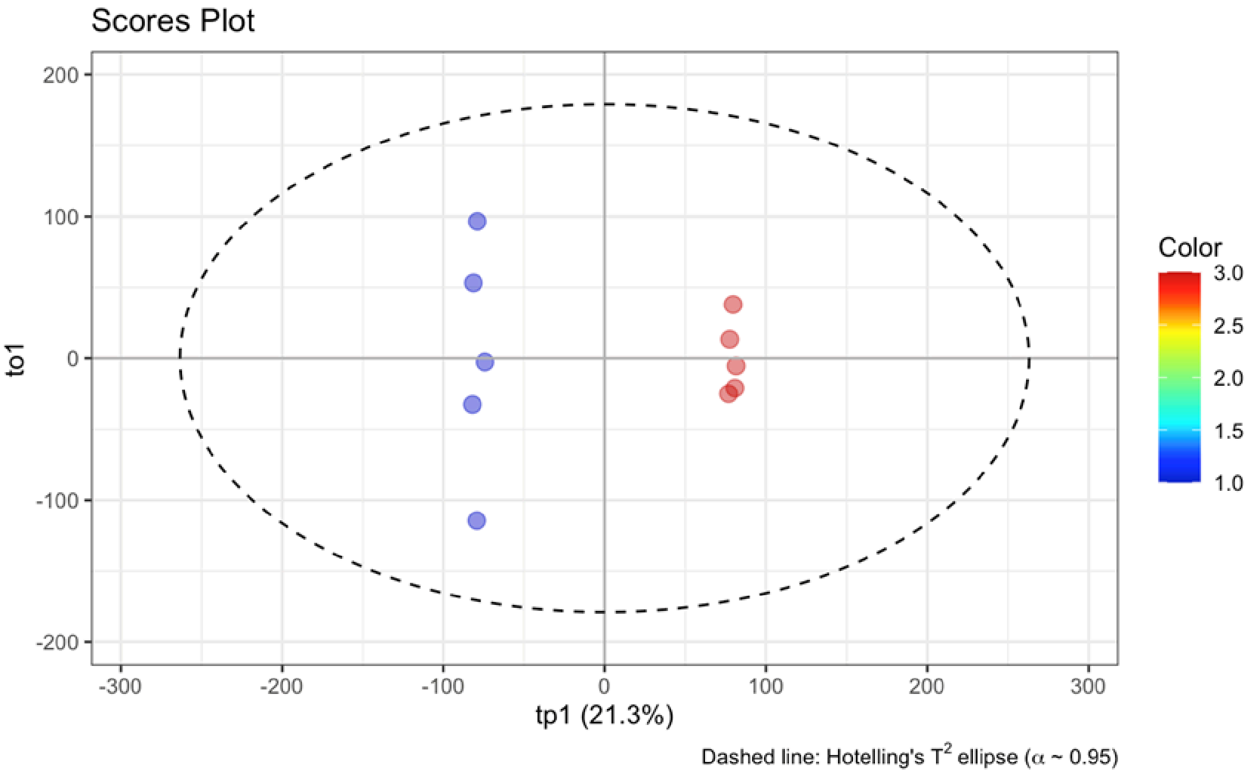

Figure S17: OPLS-DA loadings plot for 120 h post dose showing spectral regions δ_H 0.50-9.50

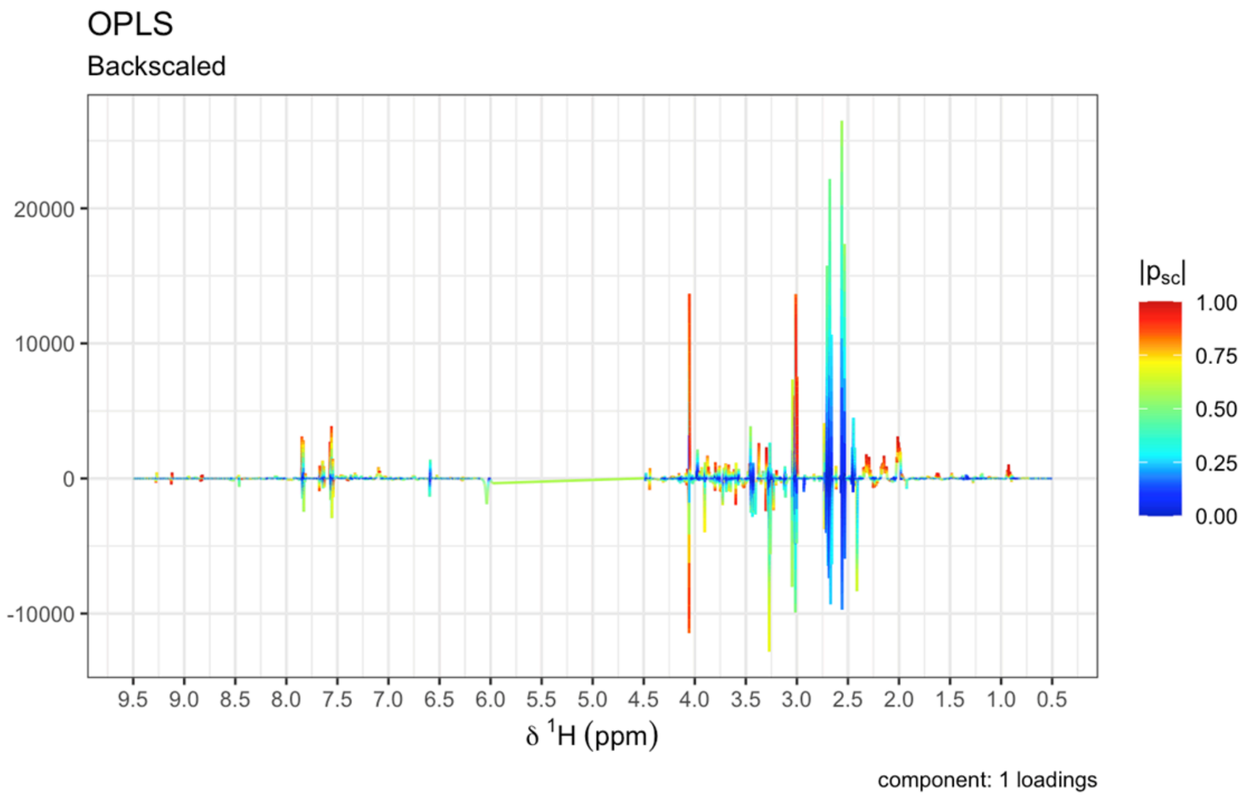

Figure S18: OPLS-DA scores plot (blue) versus 650 mg/kg (red) at 144 h post dose prior to MAA resonance excision

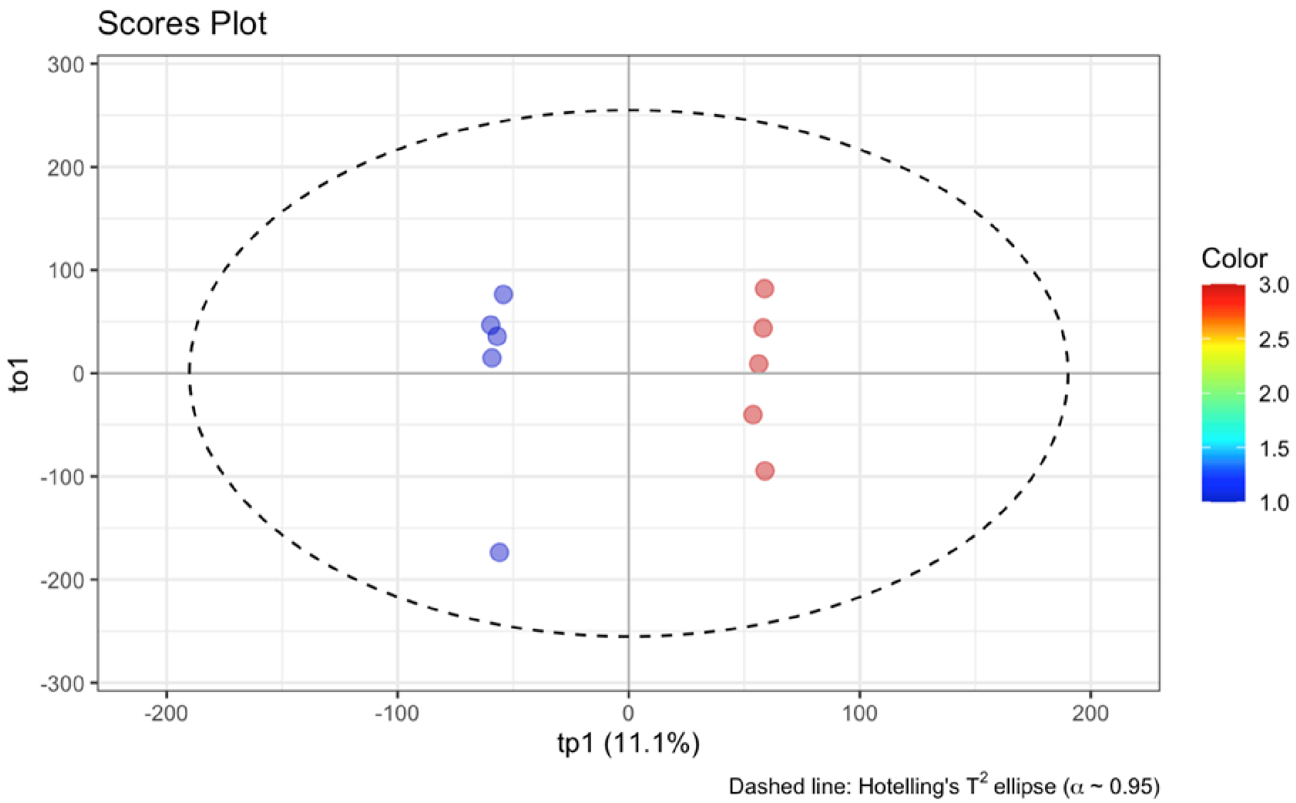

Figure S19: OPLS-DA loadings plot for 144 h post dose showing spectral regions δ_H 0.50-9.50

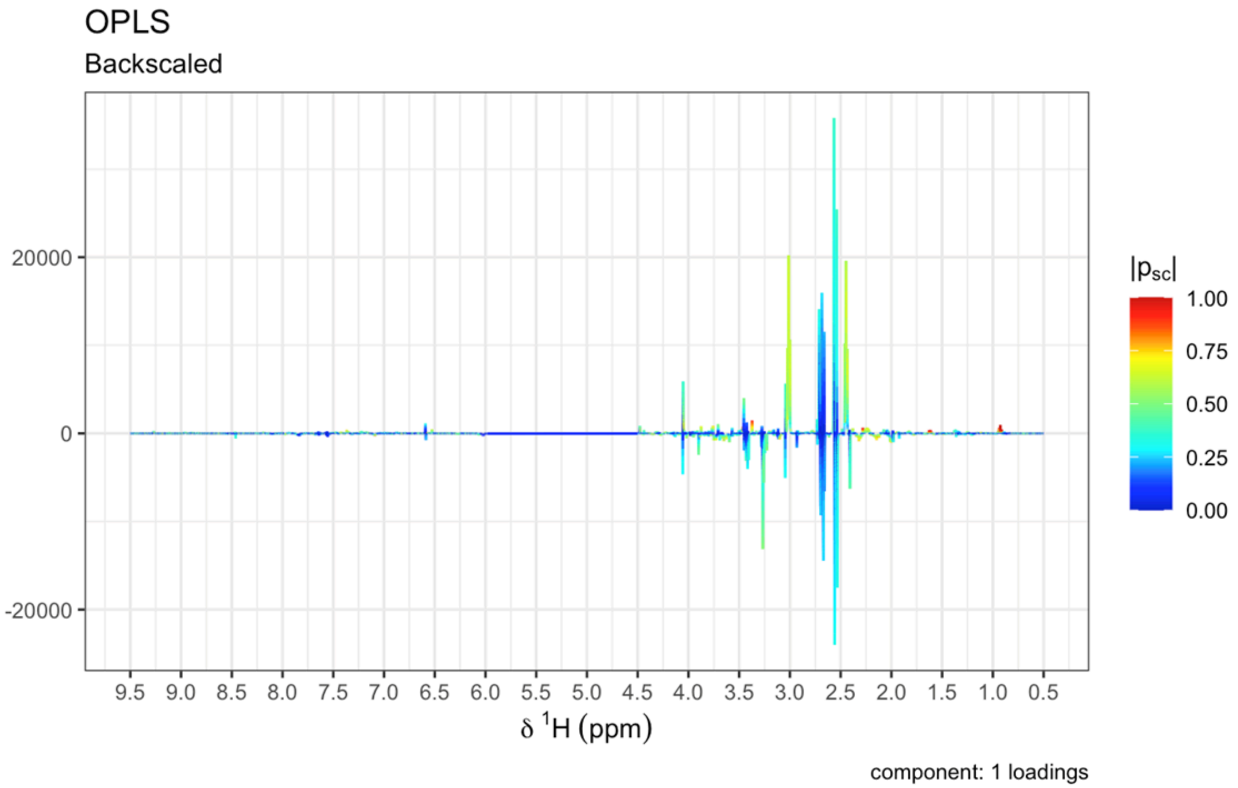

Figure S20: OPLS-DA scores plot (blue) versus 650 mg/kg (red) at 168 h post dose prior to MAA resonance excision

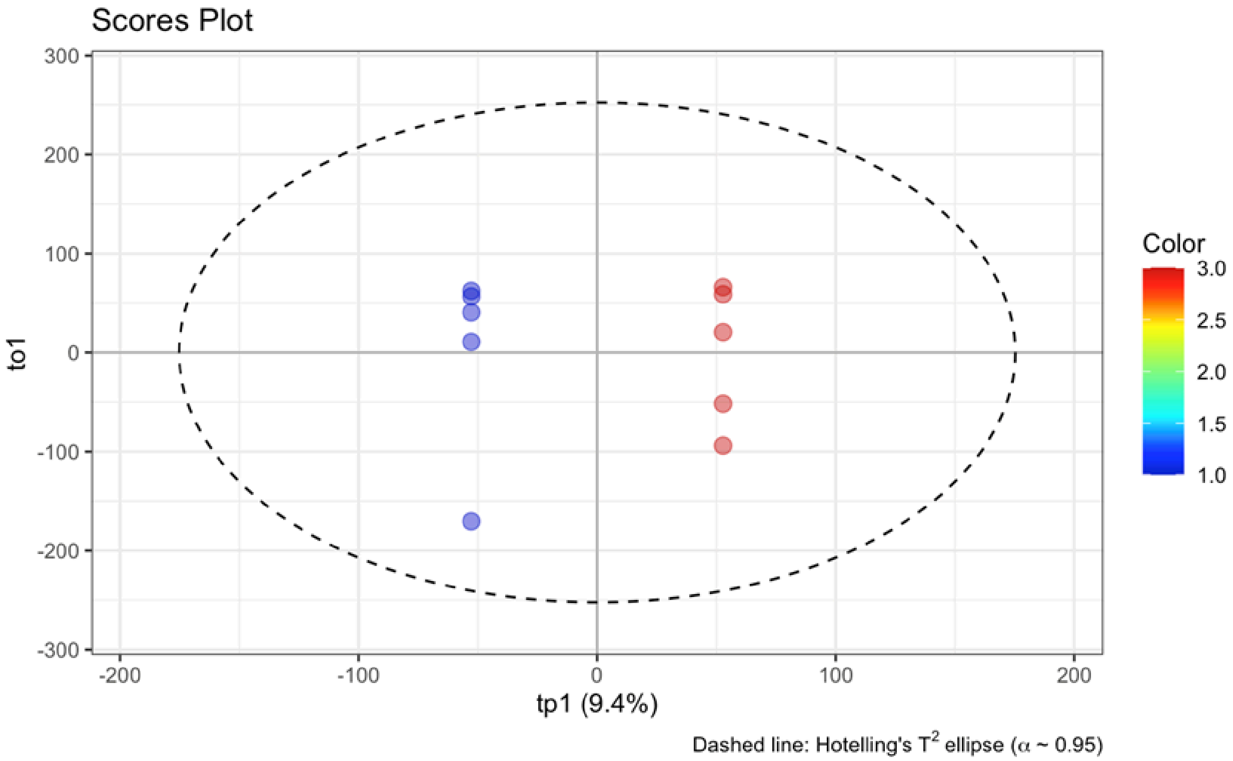

Figure S21: OPLS-DA loadings plot for 168 h post dose showing spectral regions δ_H 0.50-9.50

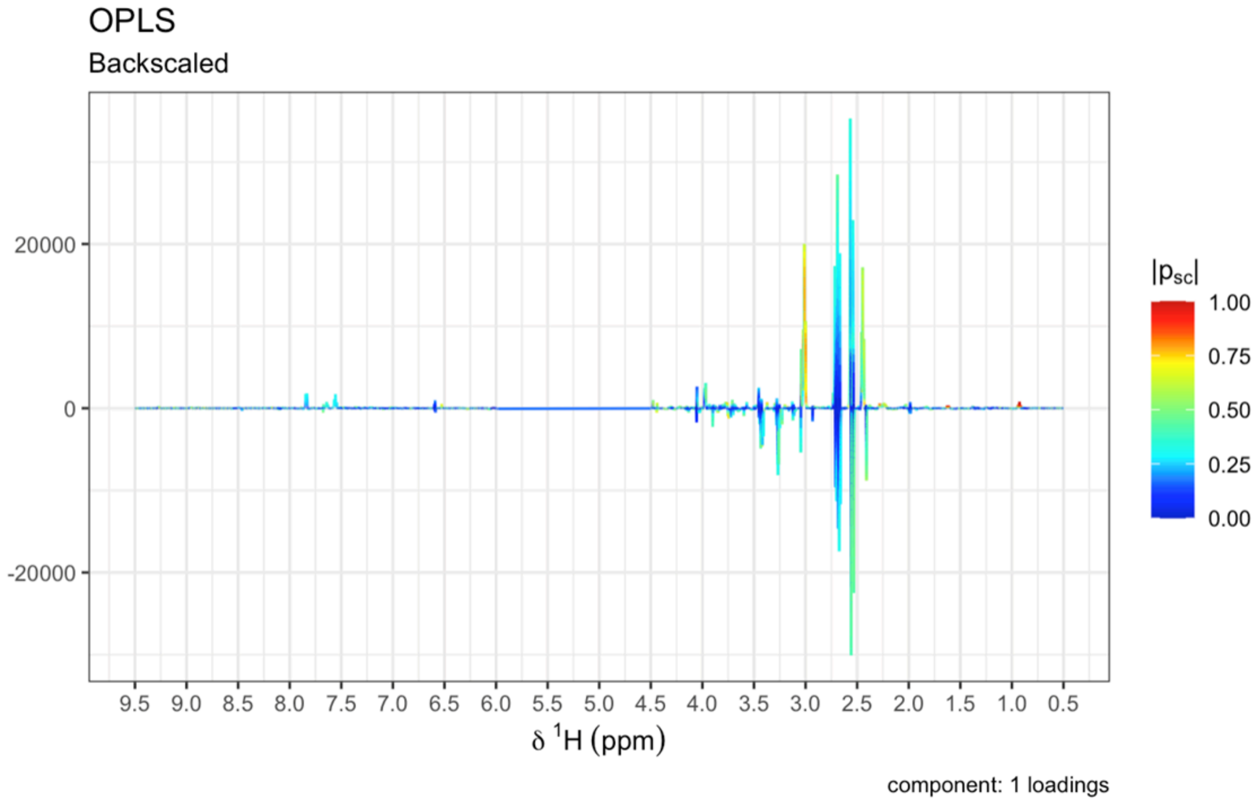

Figure S22: OPLS-DA scores plot of control (black) versus 650 mg/kg (red) at 24h post dose following MAA resonance excision

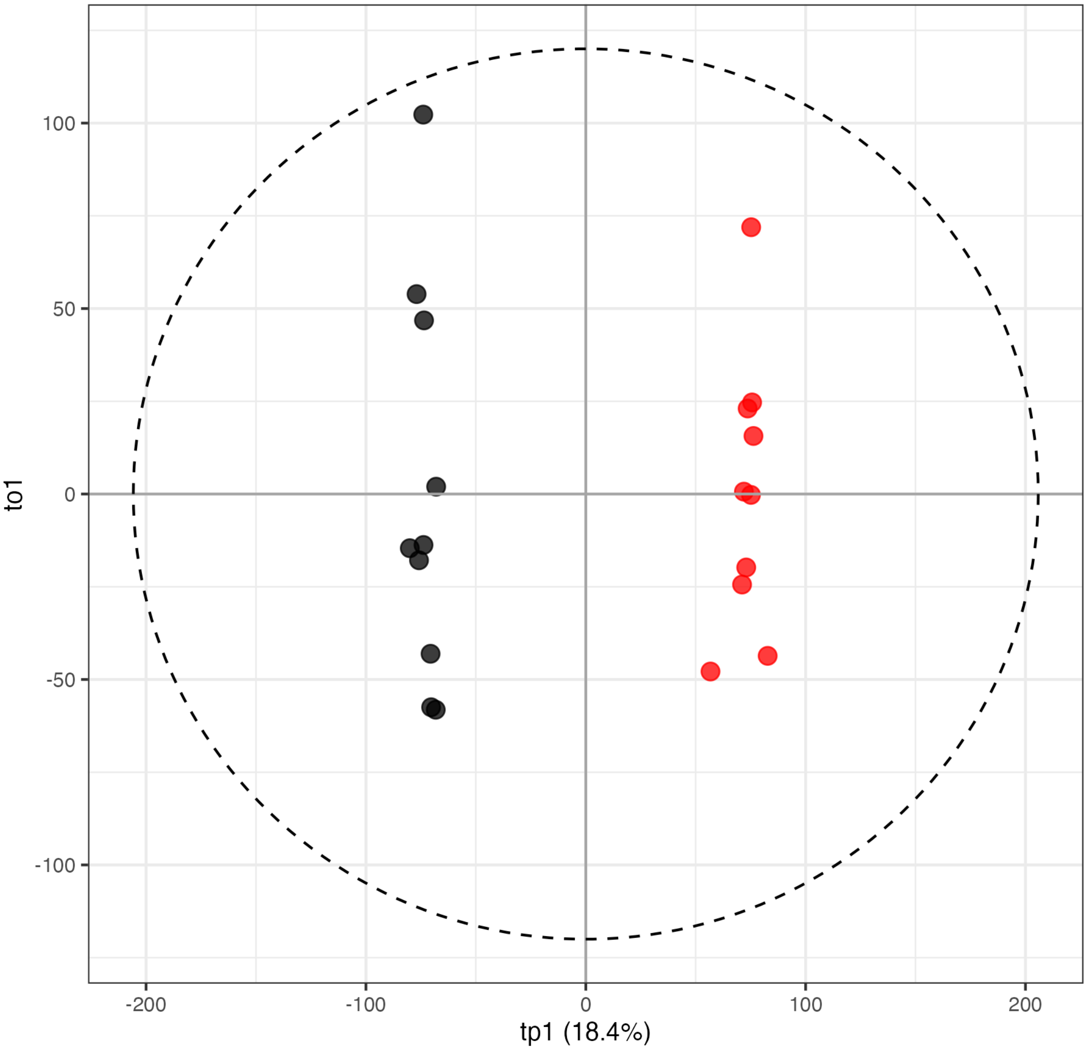

Figure S23: OPLS-DA loadings plot for 24 h post dose showing spectral regions δ_H 0.50-2.30 and δ_H 2.25-3.25 following MAA resonance excision

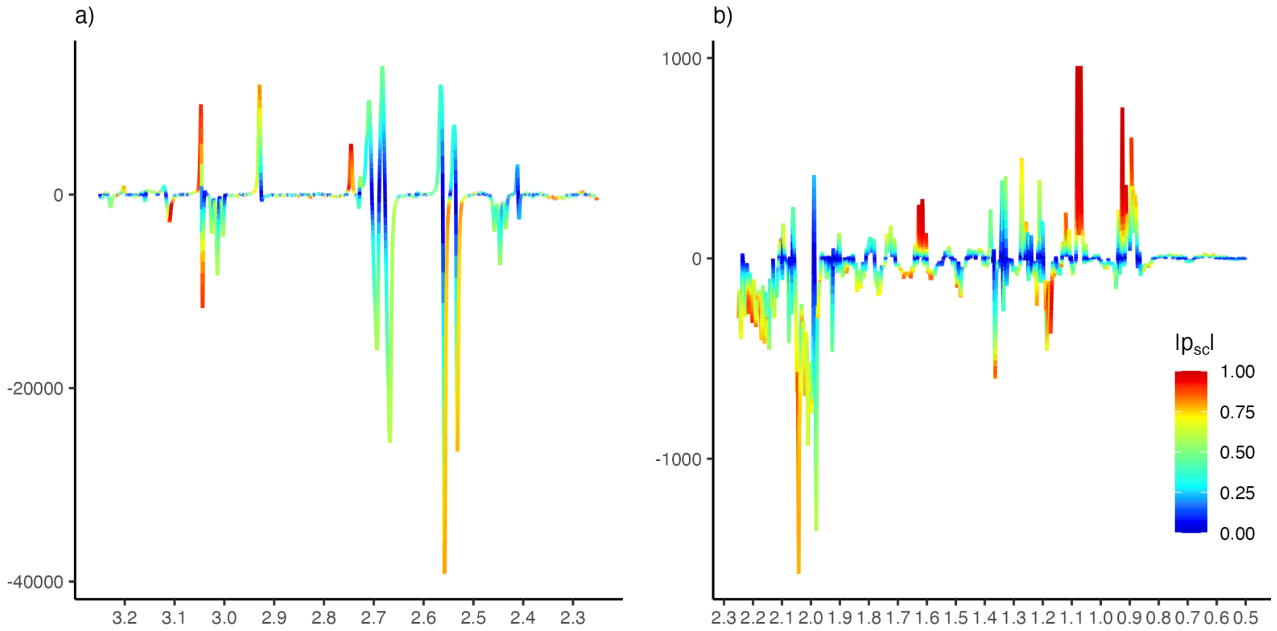

Figure S24: ^{1}H NMR spectrum of *N*-butyryl glycine authentic reference standard (600 MHz, 90:10 H_2O:D_2O)

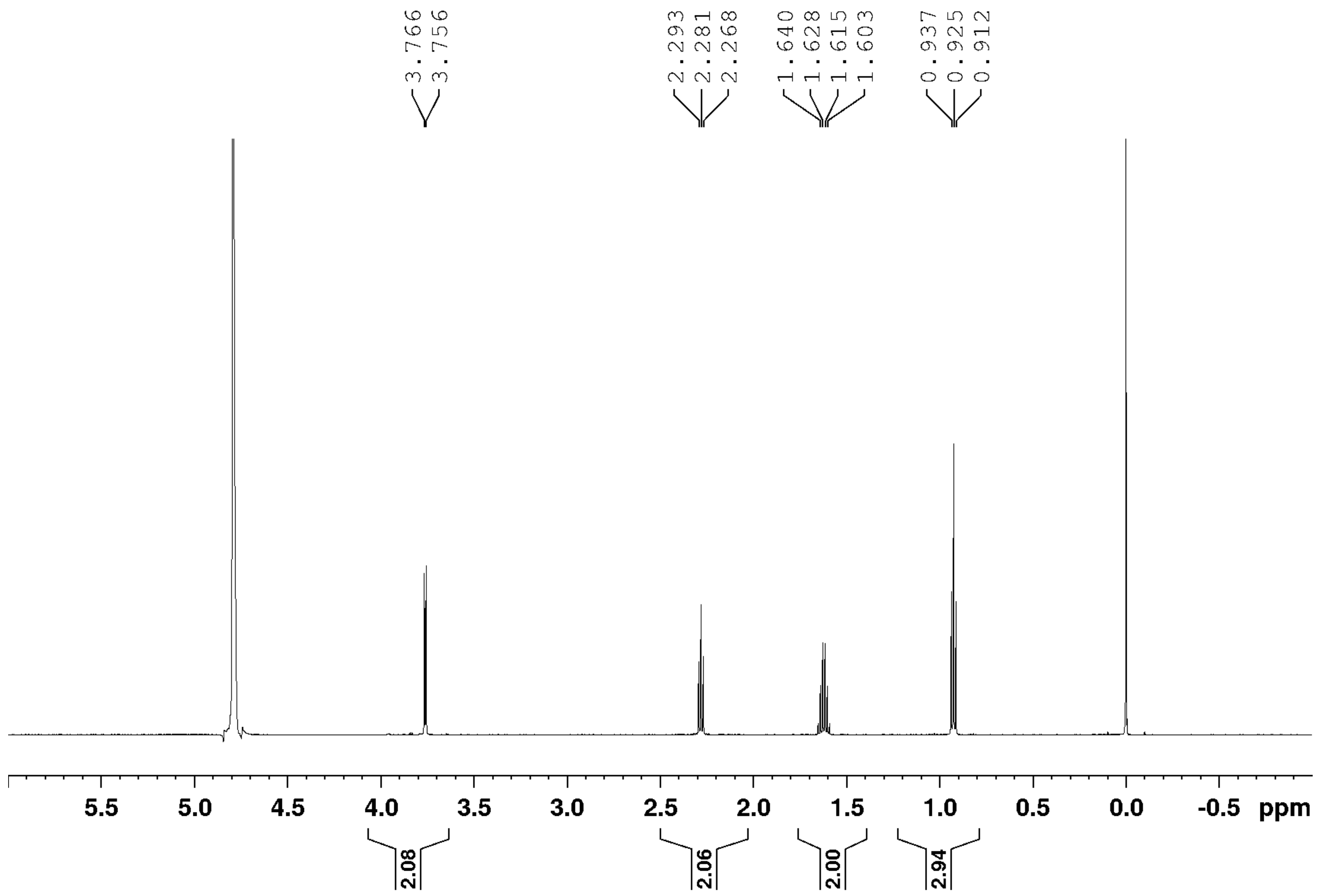

Figure S25: JRES spectrum of *N*-butyryl glycine authentic reference standard (600 MHz, 90:10 H_2O:D_2O)

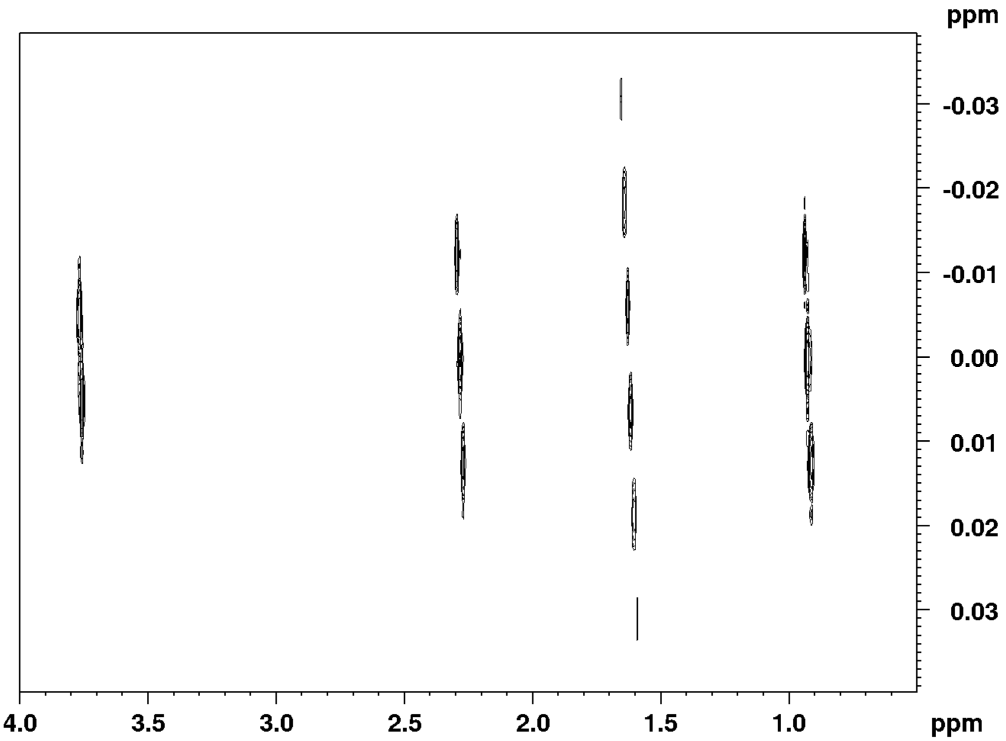

Figure S26: Stack plot of median spectra for all time points showing spectral region δ_H 0.80-1.00 and δ_H 1.50-1.70

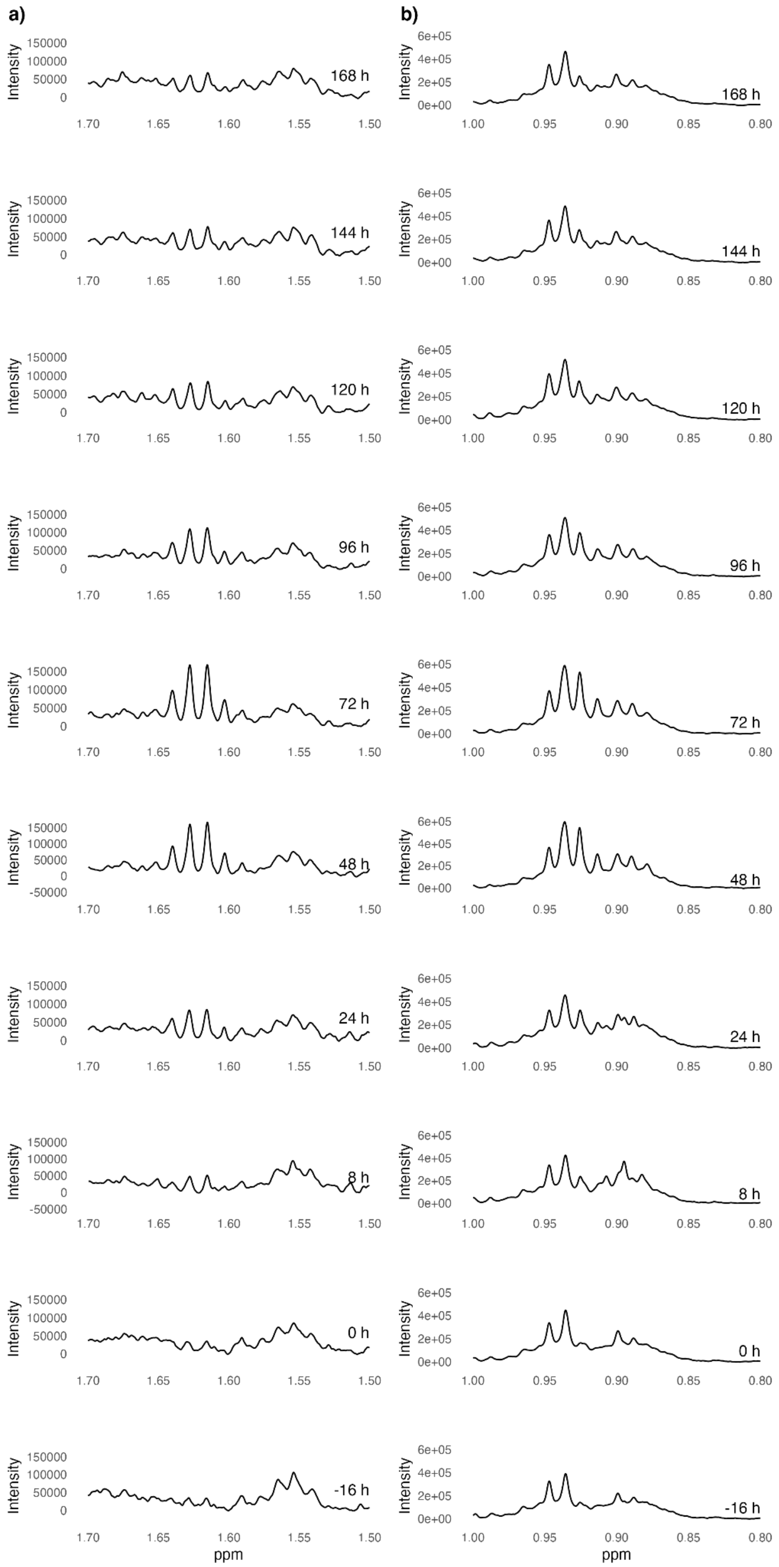

Figure S27: Dimethylamine longitudinal excretion profile

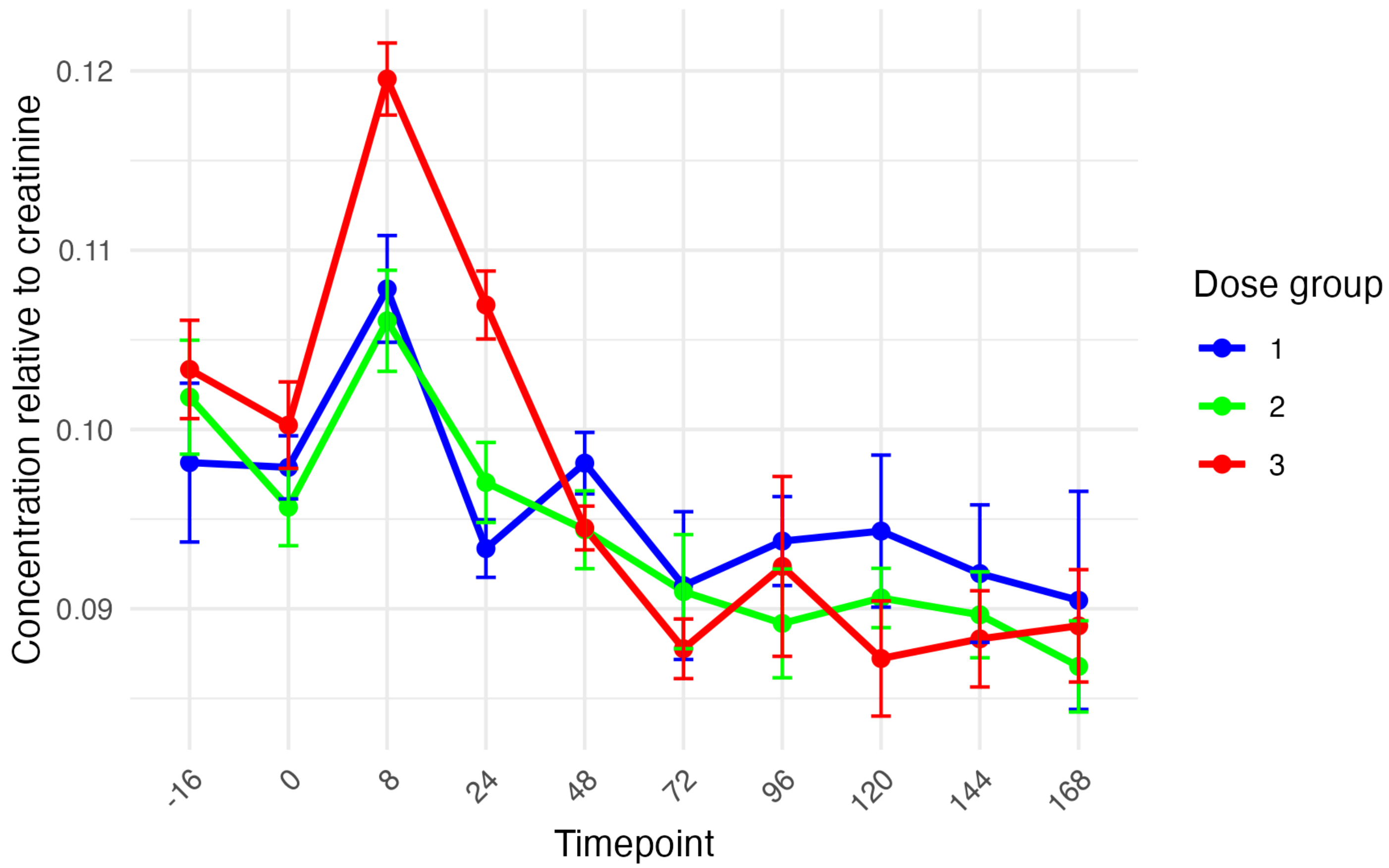

Figure S28: Dimethylglycine longitudinal excretion profile

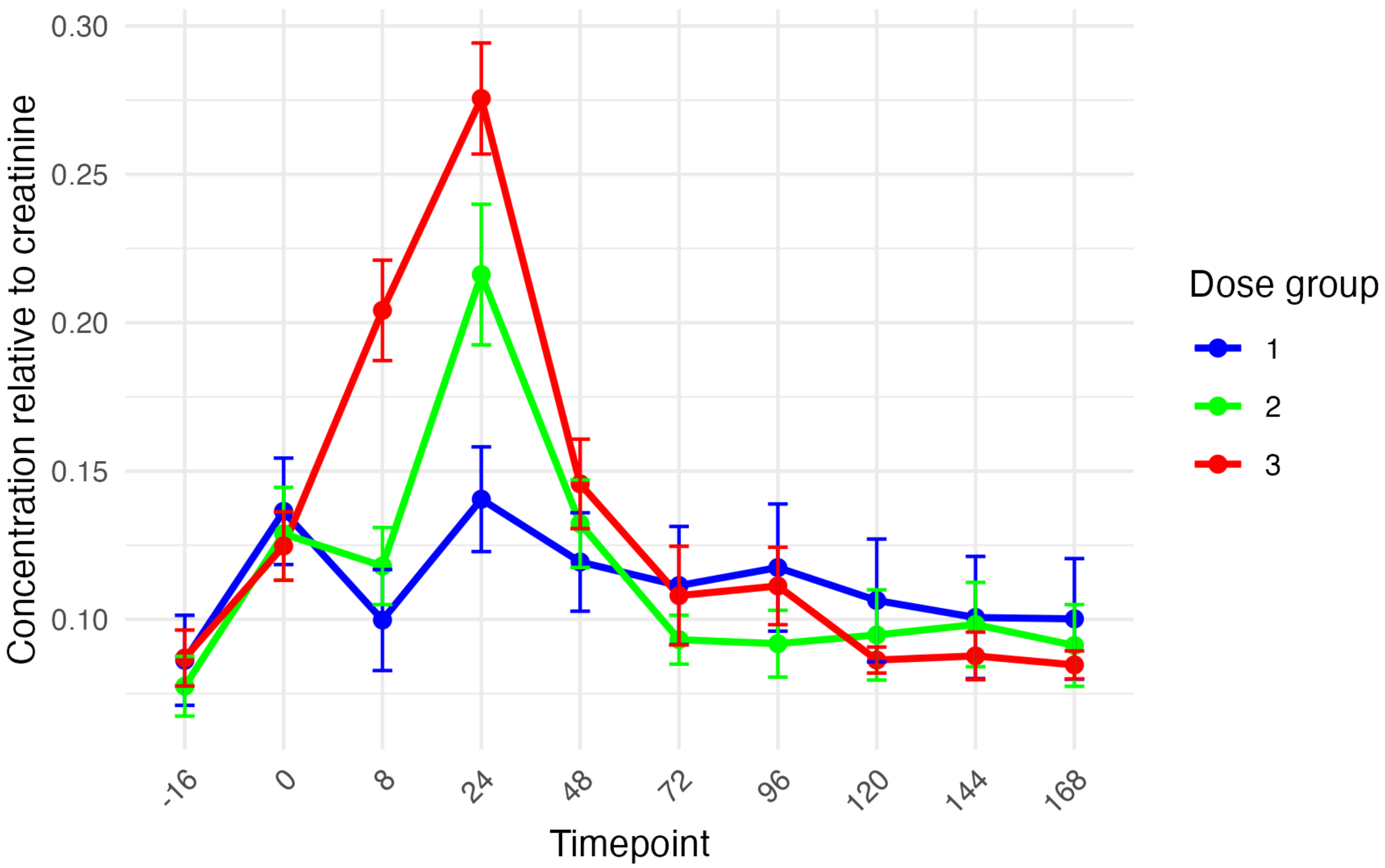

Figure S29: Hippurate Longitudinal excretion profile

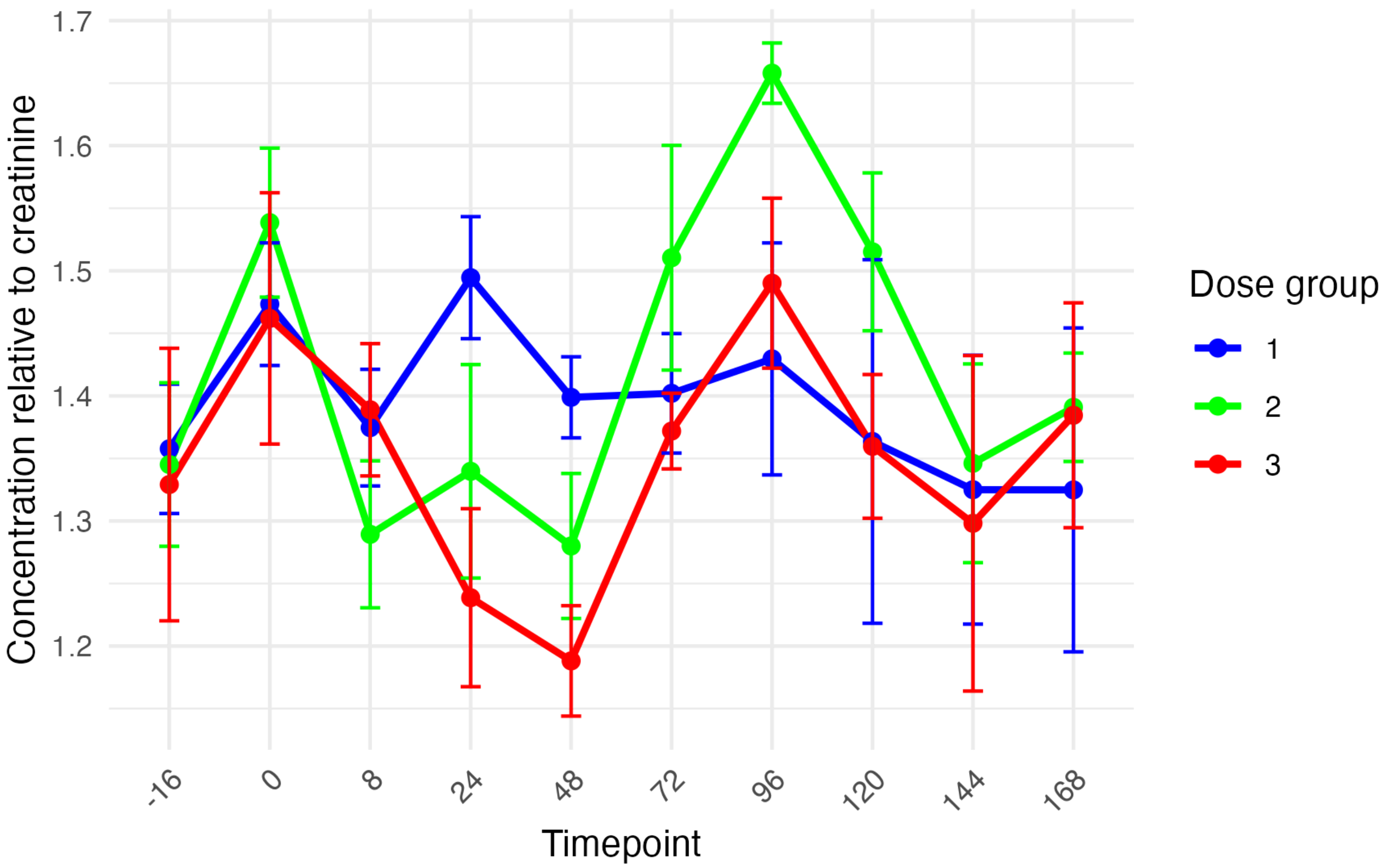

Figure S30: Ketoleucine Longitudinal excretion profile

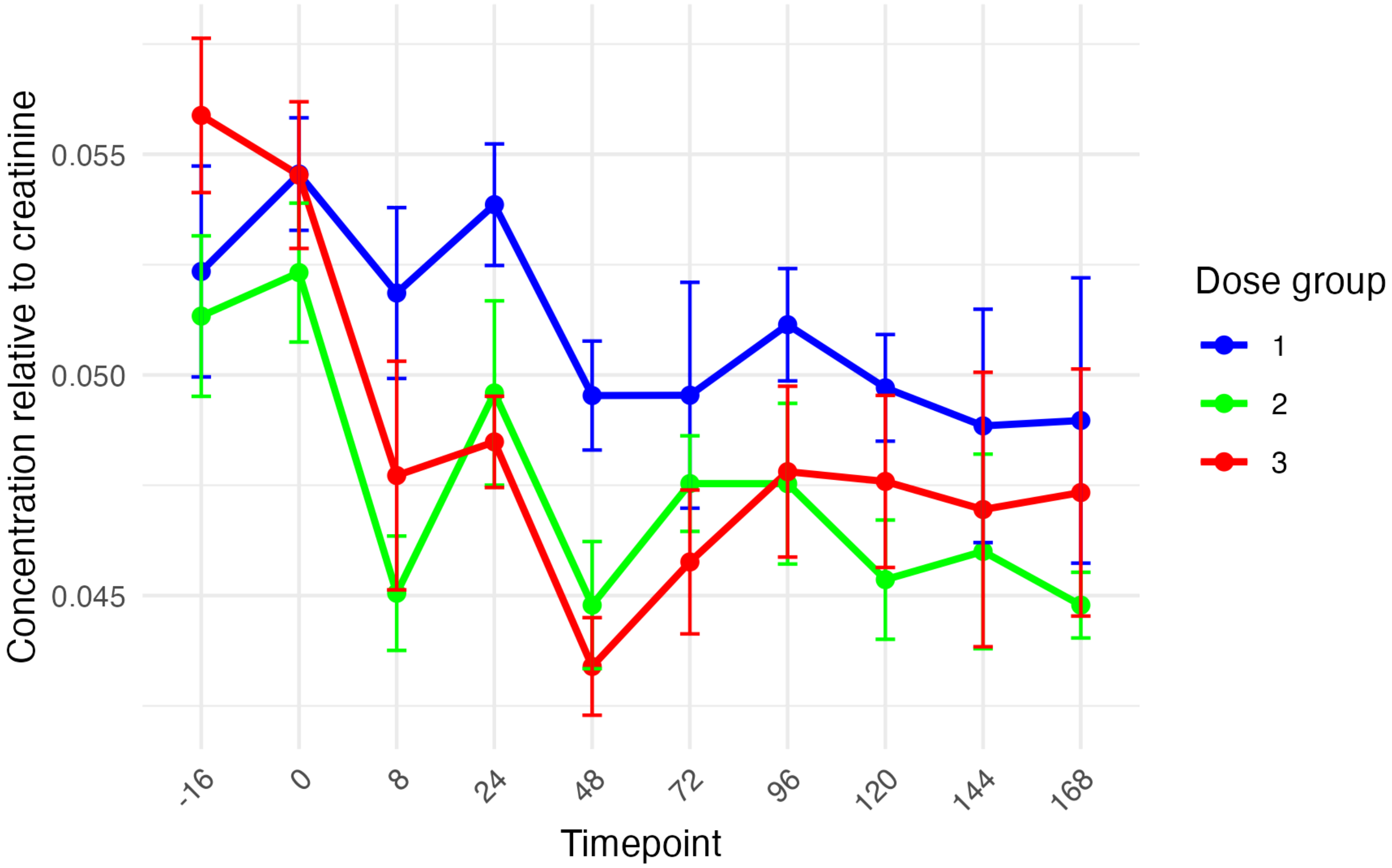

Figure S31: Alanine Longitudinal excretion profile

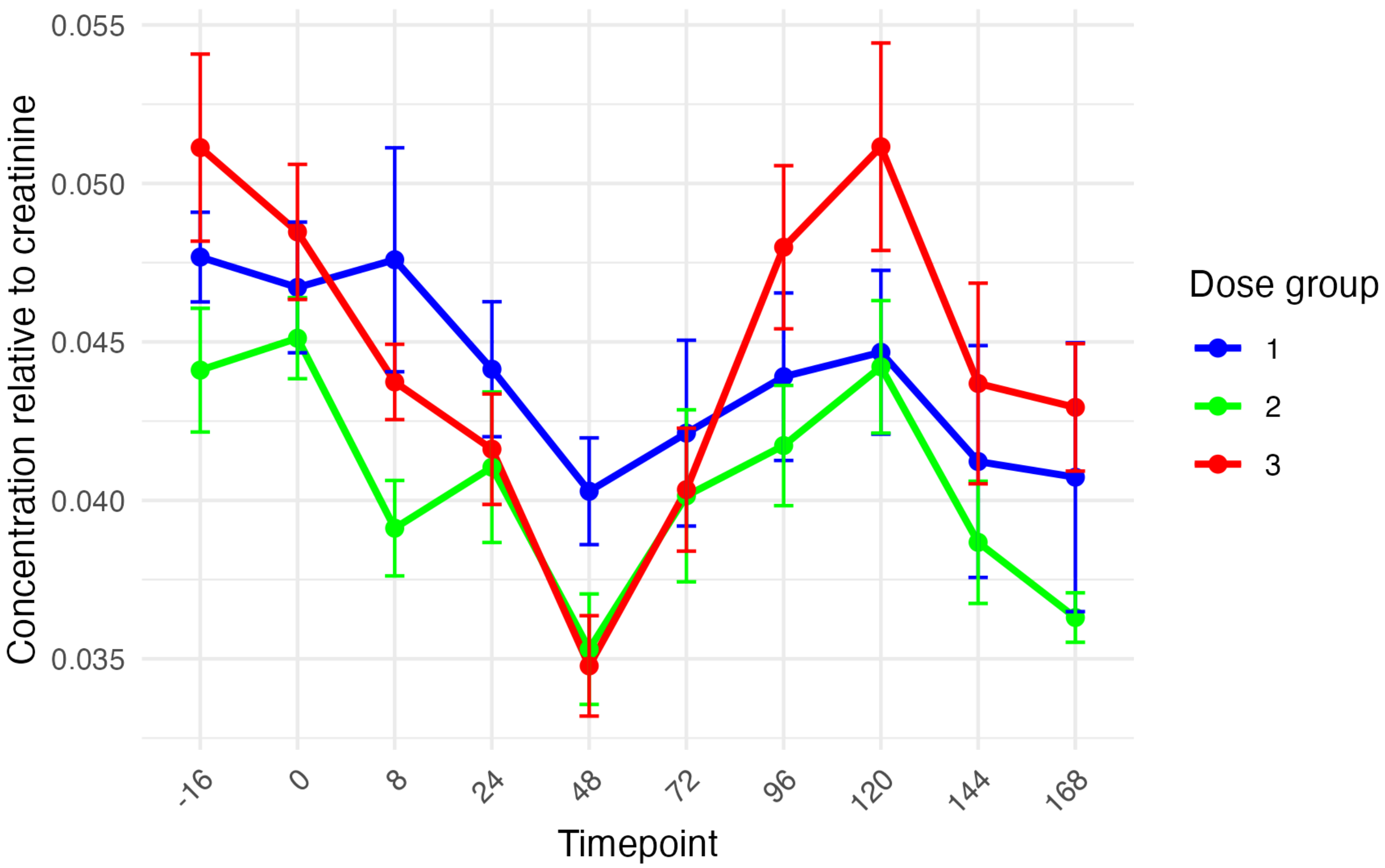

Figure S32: Acetate Longitudinal excretion profile

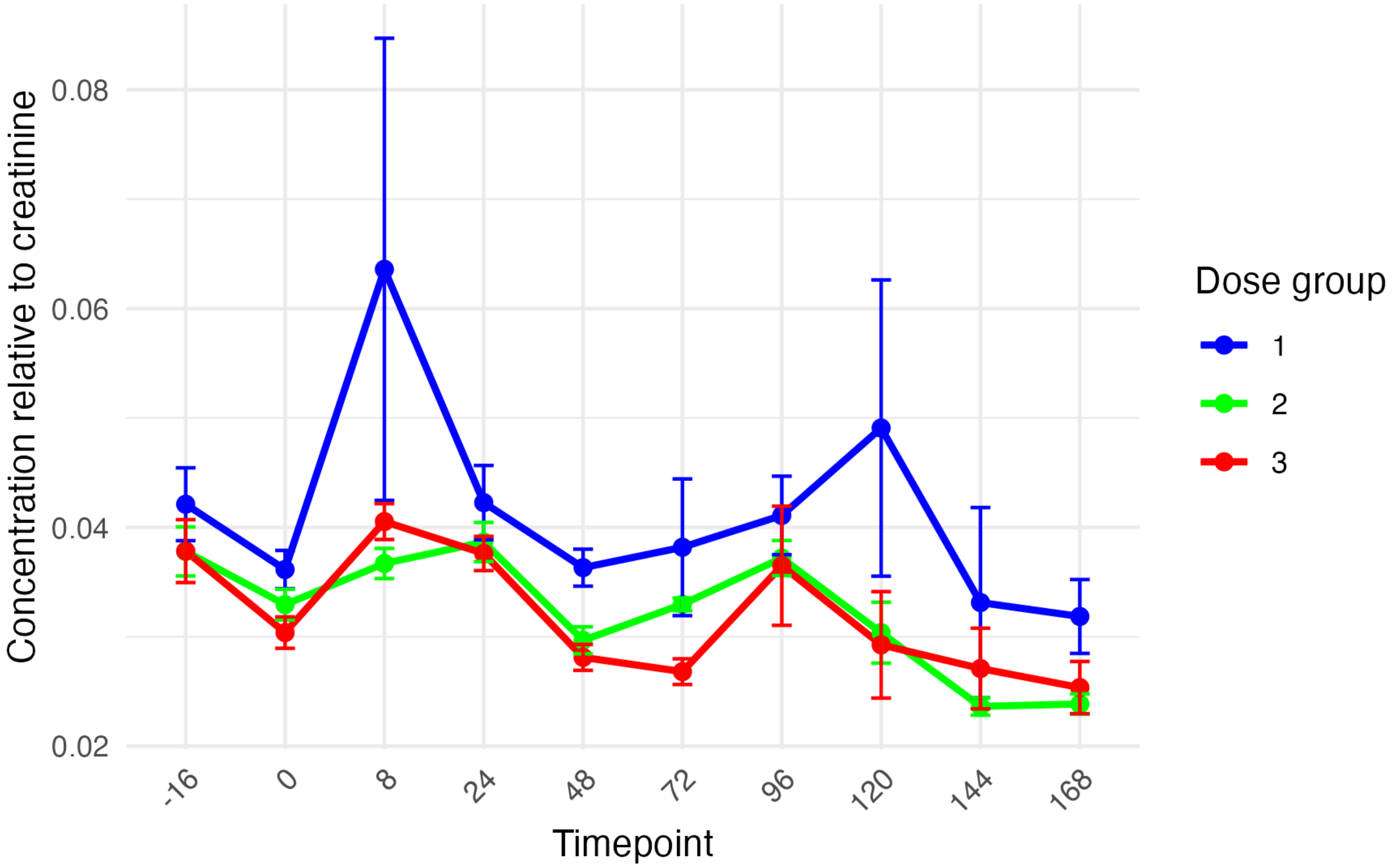

Figure S33: Taurine Longitudinal excretion profile

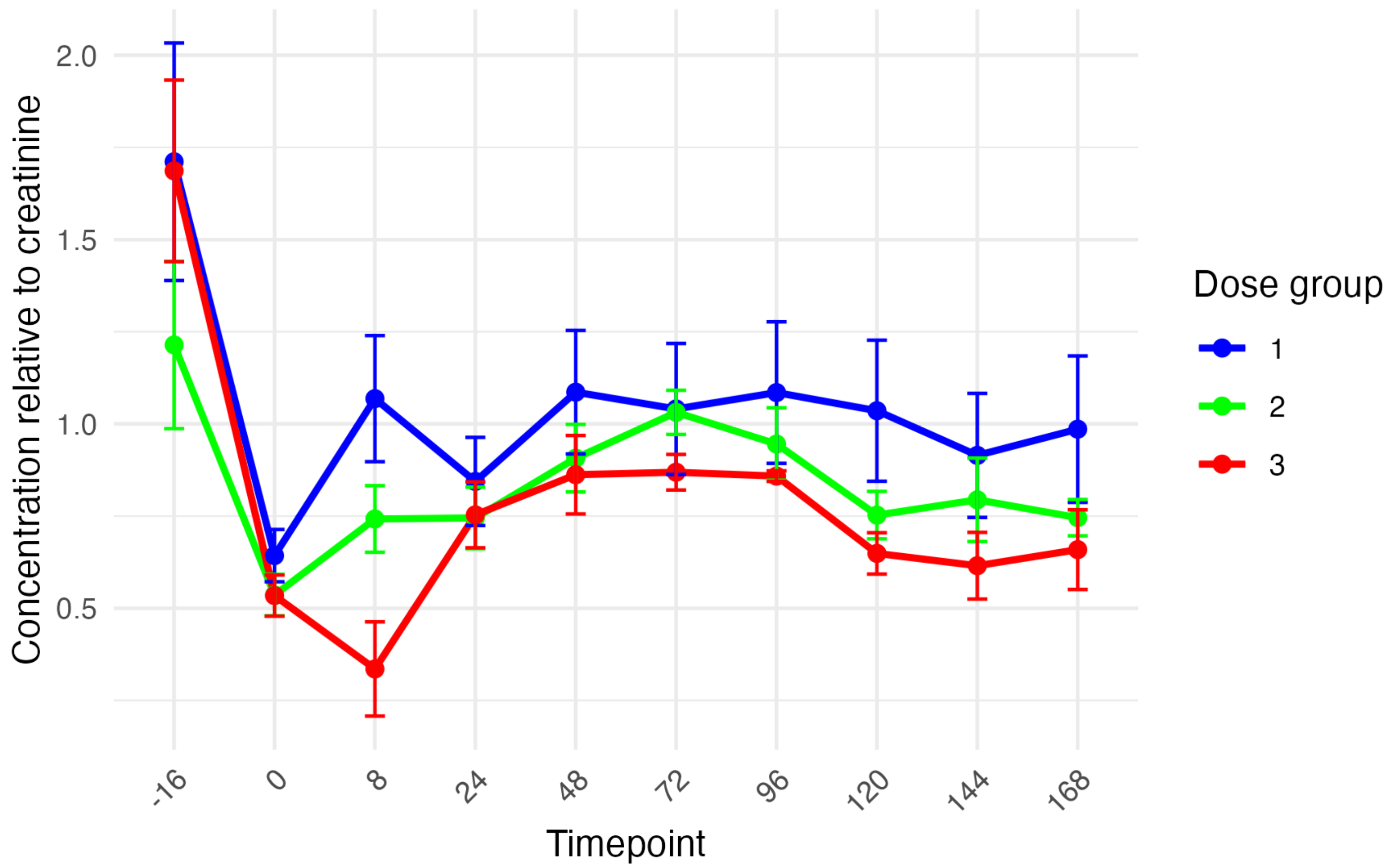

Figure S34: Glycine Longitudinal excretion profile

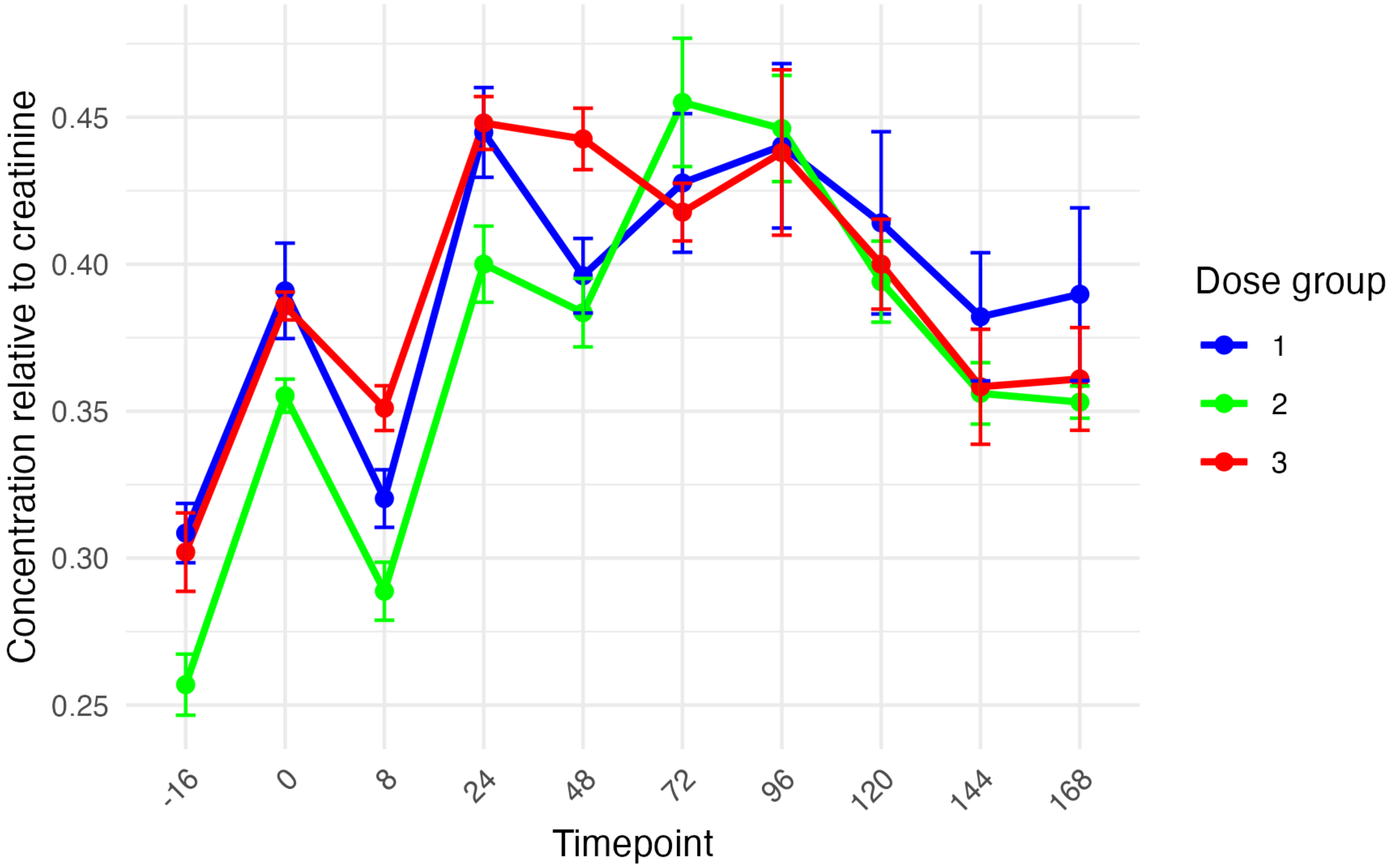

Figure S35: Phenylacetylglycine Longitudinal excretion profile

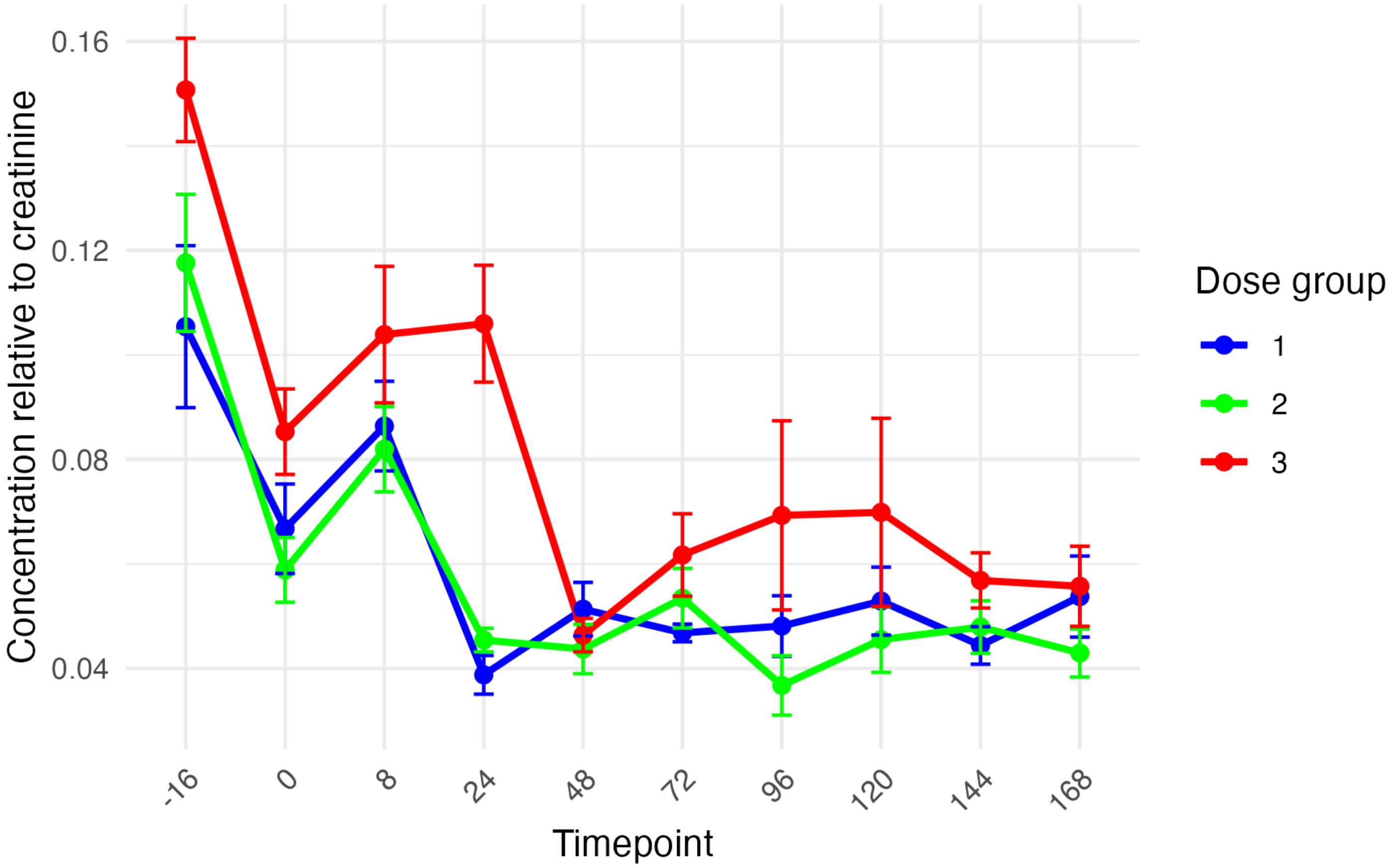

Figure S36: Allantoin Longitudinal excretion profile

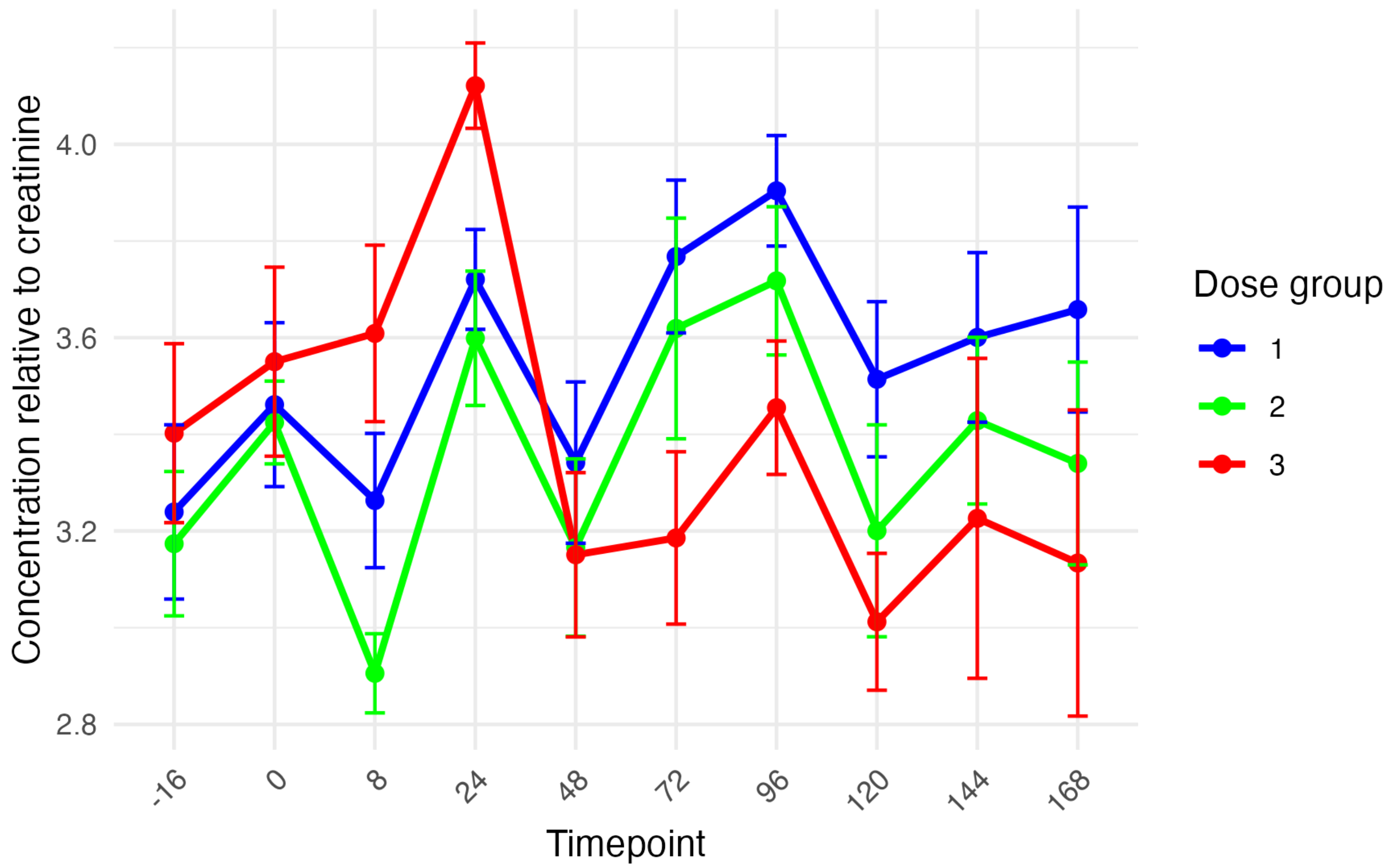

Figure S37: Formate Longitudinal excretion profile

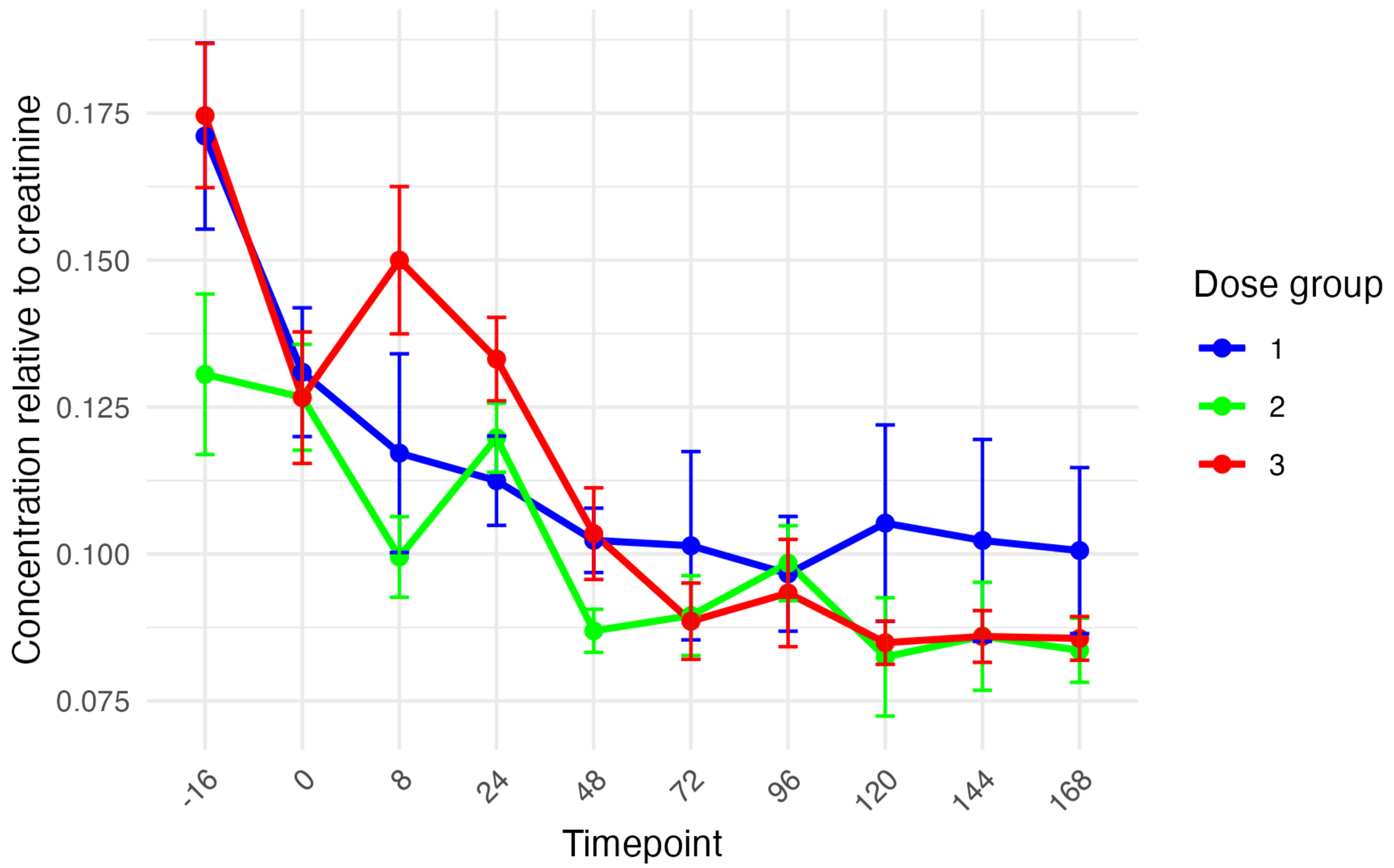

Figure S38: Trigonelline Longitudinal excretion profile

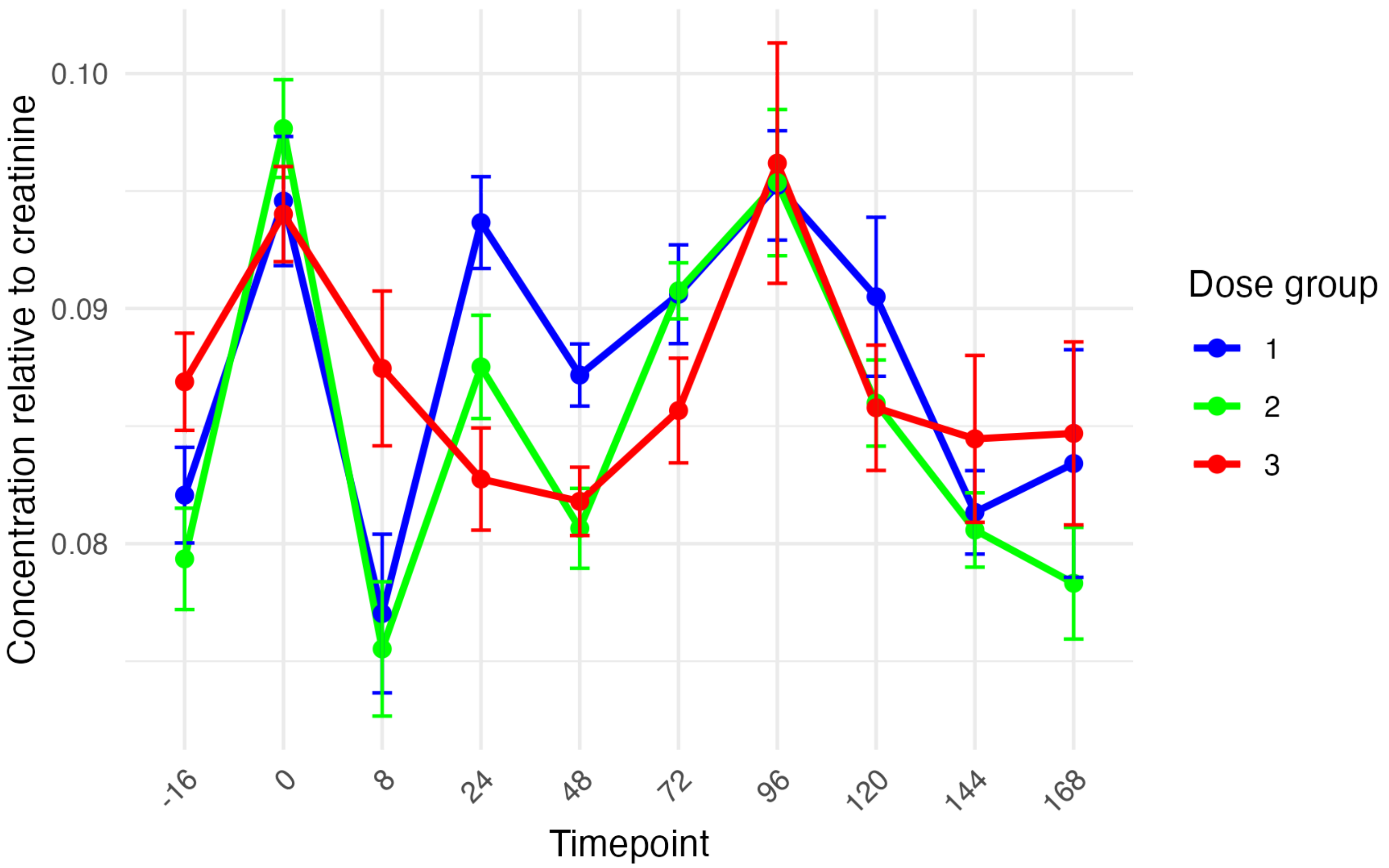

Figure S39: *N*-methylnicotinamide Longitudinal excretion profile

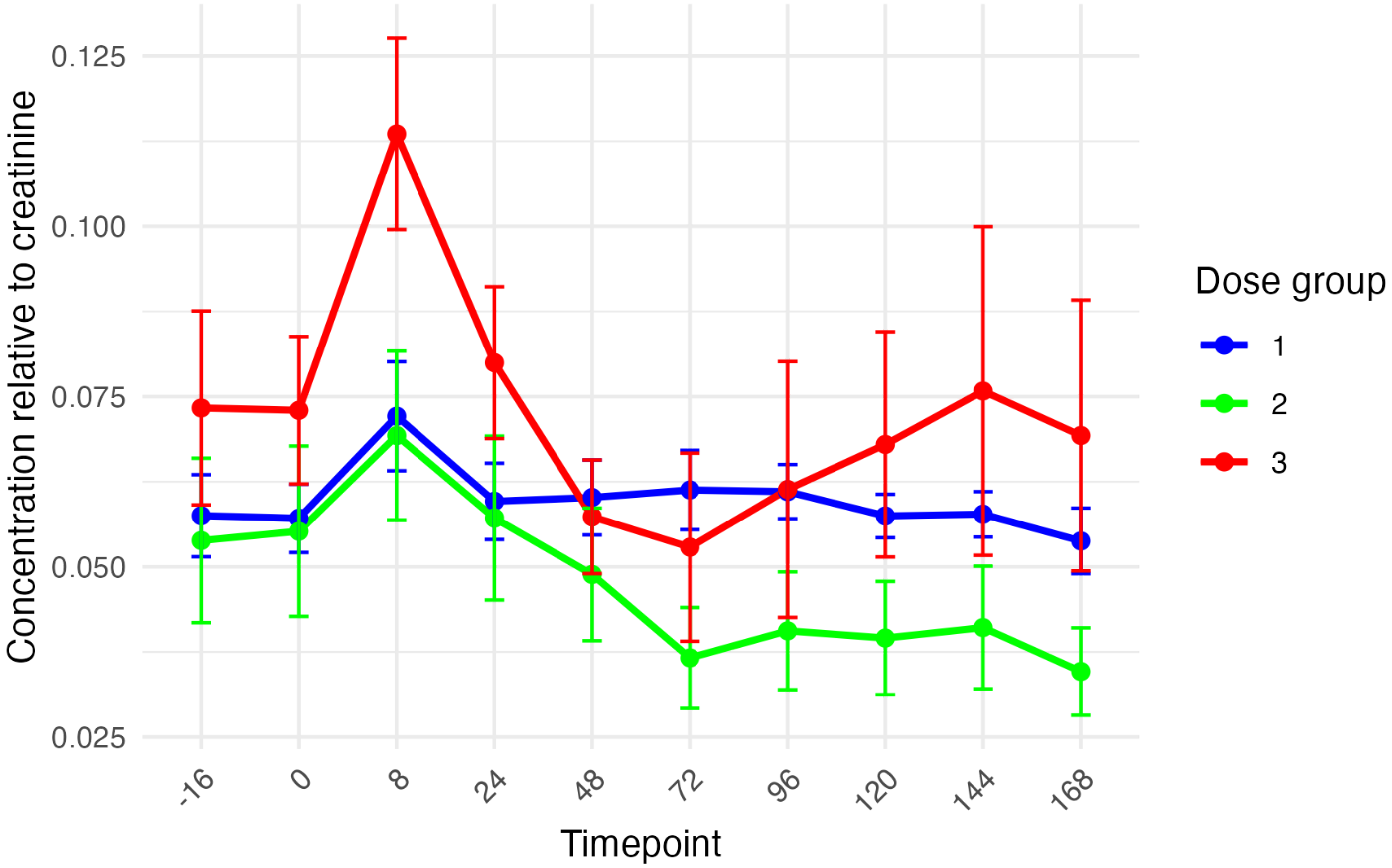

Supplement: Supplementary file 1 — Supplementary Material 1 [file 204_2026_4330_MOESM1_ESM.pdf]
